# Transcriptomic and lipid profiling analysis reveals a functional interplay between testosterone and growth hormone in hypothyroid liver

**DOI:** 10.3389/fendo.2023.1266150

**Published:** 2023-12-08

**Authors:** Leandro Fernández-Pérez, Borja Guerra, Carlota Recio, Juan José Cabrera-Galván, Irma García, Juan Vladimir De La Rosa, Antonio Castrillo, Diego Iglesias-Gato, Mario Díaz

**Affiliations:** ^1^ Instituto Universitario de Investigaciones Biomédicas y Sanitarias (IUIBS), Farmacología Molecular y Traslacional, Universidad de Las Palmas de Gran Canaria, Las Palmas de Gran Canaria, Spain; ^2^ Unidad de Biomedicina del Instituto Universitario de Investigaciones Biomédicas y Sanitarias (IUIBS) Asociada al Instituto de Investigaciones Biomédicas “Alberto Sols”, Consejo Superior de Investigaciones Científicas (CSIC)-Universidad Autónoma de Madrid, Las Palmas de Gran Canaria, Spain; ^3^ Departmento de Física Básica, Grupo de Fisiología y Biofísica de Membranas, Universidad de La Laguna, La Laguna, Spain; ^4^ Instituto de Investigaciones Biomédicas “Alberto Sols”, Consejo Superior de Investigaciones Científicas (CSIC), Centro Mixto CSIC-Universidad Autónoma de Madrid, Madrid, Spain; ^5^ Novo Nordisk Foundation Center for Protein Research (CPR), Faculty of Health and Medical Sciences, University of Copenhagen, Copenhagen, Denmark

**Keywords:** hypothyroidism, testosterone, GH, liver, transcriptome, lipids

## Abstract

Preclinical and clinical studies suggest that hypothyroidism might cause hepatic endocrine and metabolic disturbances with features that mimic deficiencies of testosterone and/or GH. The absence of physiological interactions between testosterone and GH can be linked to male differentiated liver diseases. Testosterone plays relevant physiological effects on somatotropic-liver axis and liver composition and the liver is a primary organ of interactions between testosterone and GH. However, testosterone exerts many effects on liver through complex and poorly understood mechanisms. Testosterone impacts liver functions by binding to the Androgen Receptor, and, indirectly, through its conversion to estradiol, and cooperation with GH. However, the role of testosterone, and its interaction with GH, in the hypothyroid liver, remains unclear. In the present work, the effects of testosterone, and how they impact on GH-regulated whole transcriptome and lipid composition in the liver, were studied in the context of adult hypothyroid-orchiectomized rats. Testosterone replacement positively modulated somatotropic-liver axis and impacted liver transcriptome involved in lipid and glucose metabolism. In addition, testosterone enhanced the effects of GH on the transcriptome linked to lipid biosynthesis, oxidation-reduction, and metabolism of unsaturated and long-chain fatty acids (FA). However, testosterone decreased the hepatic content of cholesterol esters and triacylglycerols and increased fatty acids whereas GH increased neutral lipids and decreased polar lipids. Biological network analysis of the effects of testosterone on GH-regulated transcriptome confirmed a close connection with crucial proteins involved in steroid and fatty acid metabolism. Taken together, this comprehensive analysis of gene expression and lipid profiling in hypothyroid male liver reveals a functional interplay between testosterone and pulsed GH administration.

## Introduction

Testosterone (T) and its cooperation with somatotropic-liver axis have a major influence on fuel homeostasis (1–4) and male-differentiated phenotype in the liver (5). In addition, T maintains fuel homeostasis via its direct binding to Androgen Receptor (AR), or after its conversion to 5α-dihydrotestosterone (DHT) (1, 2, 6, 7) and, indirectly, when it is converted to estrogen, via Estrogen Receptor (ER) (4, 8–10). Furthermore, indirect effects of T could result from regulation of liver ERα expression (11, 12), interaction with Growth Hormone receptor (GHR) signaling pathway (13–15), and its influence to support a male pattern of pituitary GH secretion that, in turn, signals a male-differentiated metabolism (5).

The physiological role of AR in hepatic lipid metabolism has been shown in the context of hepatic AR knockout male mice (6). AR knockout mice developed an increased liver lipogenesis and steatosis with upregulation of genes involved in fatty acid (FA) synthesis, and downregulation of genes involved in fat oxidation. Similarly, a reduction of DHT synthesis in 5α-reductase type 1 deficient, or administration of a 5α-reductase type 1 inhibitor to male obese Zucker rats, also caused fatty liver disease (16). Otherwise, T signaling deficiency in male rats enhanced diet-induced liver fat accumulation (17). In men, T lowering is associated with important clinical lipid-related disorders (i.e., dyslipidemia, atherosclerosis, diabetes, metabolic syndrome). In contrast, T supplementation therapy in hypogonadal men improved lipid profiling by lowering cholesterol, blood sugar and insulin resistance (4, 17, 18). The relevance of T aromatization to maintain glucose tolerance and insulin sensitivity via estradiol (E2)-ERα signaling, has been described in both male and female aromatase knockout mice where the estrogen biosynthetic enzyme aromatase was inactivated (8). Accordingly, aromatase knockout mice showed reduced glucose oxidation, and increased adiposity and insulin levels, and E2 replacement restored glucose tolerance and insulin sensitivity through activation of liver ERα (19–21). The aromatase knockout male mice also developed fatty liver and insulin resistance in parallel to increased hepatic FAs uptake and *de novo* lipogenesis (22). Notably, aromatase-deficient men also develop steatohepatitis and insulin resistance which supports a critical role of endogenous E2 in control of glucose and lipid homeostasis in human (9). Furthermore, the positive regulation of ERα gene expression in rat liver by *Thriyodotironine (*T3), GH and/or T (11, 12), could contribute to E2-mediated effects of these hormonal replacement.

Clinically relevant is the functional cooperation between T and GH (13–15, 23). In hypopituitary men, T and GH independently induce protein synthesis and, when administered as combinatory treatment, they exert additive effects on protein synthesis, insulin-like growth factor-I (IGF-1) gene expression and stimulation of fat oxidation. These hormone interactions contribute to enhance physiological effects on protein and energy metabolism that mainly affect the liver rather than peripheral tissues (13, 15). On the other hand, liver-associated diseases might also be connected to the absence of physiological cooperation between T and GH. This is, in part, because somatotropic-liver axis is a main regulator of intrahepatic lipid content, where GH and IGF1 are potential disease modifiers in the development and progression of lipid metabolism associated disorders such as nonalcoholic fatty liver disease (NAFLD) (24). Relevant to this study, thyroid hormones are direct and indirect modulators of hepatic lipid metabolism (25–27). Notably, hypothyroidism mimics the effects of T and GH deficiency in the liver (3) because thyroid hormones stimulate the synthesis and secretion of T (28–30) and pituitary GH (31, 32). However, the influence of T, and its cooperation with GH, on gene expression profiling and lipid composition in hypothyroid liver is still misunderstood. Hence, this study was conceived in the context of hypothyroid-orchiectomized rats to add comprehensive information about the influence of T replacement, and its interaction with pulsed GH administration, on transcriptome and lipid composition in the male liver.

## Materials and methods

### Materials

Recombinant human GH was kindly donated by Pfizer laboratories (Madrid, Spain). All regents, drugs and compounds cited in this work were purchased from Sigma-Aldrich (St. Louis, MO, USA), unless otherwise indicated.

### Animal treatment

All animal experiments were conducted in accordance with Spanish and EU animal care guidelines and with approved protocol by the Animal Care and Use Committee of the University of Las Palmas de Gran Canaria (OEBA-ULPGC 2006/07824). Adult (2-3 months of age) male Sprague-Dawley rats (n=6 per group) used throughout the study were housed four or five to a cage, maintained under standard laboratory conditions (12-hour light/dark cycle and controlled temperature; 21-23 °C) with food (standard chow diet, A04 SAFE Panlab, Barcelona, Spain) and water provided *ad libitum*, at ULPGC Animal Unit. To generate the hypothyroid experimental model, rats were provided with 0.05% (w/v) methimazole (MMI) in drinking water *ad libitum* for 5 weeks (equivalent to a MMI dose of 50-60 mg/kg/day) starting on postnatal day (PND) 59 until sacrifice on PND94 (33). Calcium chloride (1%) was included with MMI in the water to ensure adequate dietary calcium intake because hypothyroidism decreases food intake by up to 40% (33). The MMI-containing water was replaced twice a week. Another group of rats were no MMI-treated during this period (INTACT). Two weeks after starting MMI administration, TX rats were orchiectomized or sham-operated to obtain TXOX or testis-intact hypothyroid (TXSO) groups, respectively. Bilateral orchiectomy using sterile procedures was performed under isoflurane anesthesia. Testicles were removed through a single medial raphe scrotal incision after transection of spermatic cords. In sham controls, the testicles were visualized but not removed. The INTACT group was also subjected to sham-surgery to provide testis-intact euthyroid group (INTACTSO) (**Figure S1A**). Four days after orchiectomy, we began hormonal replacement with TP (50 μg/kg; sc; 5 days per week) (TXOXTP) or vehicle (0.20 ml of corn oil; VEH) (TXOX) for 20 days before hormonal replacement with GH (TXOXTPGH and TXOXGH, respectively) or T3 (TXOXTPT3 and TXOXT3, respectively), T3 plus GH (TXOXT3GH) or VEH (TXOXTP) for 7 days (**Figure S1B**). GH (0.3 mg/kg/day) was administered as two daily subcutaneous (sc) injections in 12-h intervals (08:00h and 20:00h) to mimic the physiological male-specific GH secretion (5). T3 (10 μg/kg body weight) was administered as a single daily intraperitoneal (ip) injection. T3 was dissolved in a minimum volume of 0.01 N NaOH and was brought up to the appropriate concentration with sterile saline. Hypothyroidism status was corroborated by measuring serum levels of thyroxine (T4) and T3 at PND94 and monitoring body weight gain every 7 days after MMI administration was started. Body weight gain was calculated every week for each animal by using the formula BWy – BWx, where BWy is body weight calculated 7 days after last measurement (BWx). On PND94, body weight gain was calculated through the determination of body weight at PND59 and PND94.

Twenty-four hours (in the case of TP or T3) or twelve hours (in the case of GH) after the last injection, animals were euthanized by isoflurane overdose. On PND94, blood samples were collected, and serum stored at -80°C until analysis. Portions of the liver were snap frozen in liquid nitrogen and stored at -80°C until processed for mRNA analysis.

### Serum analysis

Serum free T4 and T3 concentrations were measured in duplicate by enzyme immunoassay (Beckman Coulter Inc., Pasadena, CA, USA), with a detection limit of 0.60 ng/dl and 88 ng/dl, respectively. Serum levels of glucose, total cholesterol (CHO), and triacylglycerols (TG) were quantified by using an Olympus AU2700 chemistry analyzer (Beckman Coulter Inc.). Total T (ng/dl) was determined by Access Immunoassay system test kit and Unicel DXI 800 auto-analyzer (Beckman Coulter Inc.). Serum levels of leptin and IGF-I were determined by using rat immunoassays (Quantikine, R&D systems, Minneapolis, MN, USA) according to manufacturer recommendations. The IGF-I and leptin assays included quality controls provided by the manufacturer, and the standard curves of the assays were performed using the samples provided by the manufacturer. All the samples were assayed together, and each sample was assayed in duplicate.

### Hepatic lipid analysis

Total lipids were extracted with chloroform/methanol (2:1 v/v). The organic solvent was evaporated under a stream of nitrogen, and the lipid content was determined gravimetrically and stored in chloroform/methanol (2:1 v/v) containing 0.01% butylated hydroxytoluene as an antioxidant (34). Lipid classes were separated from a fraction of total lipids by one-dimensional double-development high-performance thin-layer chromatography using methyl acetate/isopropanol/chloroform/methanol/0.25% (w/v) KCl (5:5:5:2:1.8 by vol.) as the developing solvent system for the polar lipid classes and hexane/diethyl ether/acetic acid (22.5:2.5:0.25 by vol.) as the developing solvent system for the neutral lipid classes (34). Lipid classes were quantified by charring with 3% (w/v) aqueous cupric acetate containing 8% (v/v) phosphoric acid followed by calibrated scanning densitometry using a Shimadzu CS-9001PC dual-wavelength spot scanner (Shimadzu Europe, Duisburg, Germany). Total and neutral lipid fractions were subjected to acid-catalyzed transmethylation for 16 h at 50°C using 1 ml of toluene and 2 ml of 1% sulfuric acid (v/v) in methanol. The resultant FA methyl esters were purified by thin-layer chromatography and visualized under spraying with 1% iodine in chloroform (34). Fatty acid methyl esters were separated and quantified by using a Thermo gas chromatograph equipped with a flame ionization detector (250°C) and a fused silica capillary column Supelcowax™ 10 (30 m x 0.32 mm I.D.) (Sigma Aldrich, St. Louis, MO, USA). Individual fatty acid methyl esters were identified by referring to authentic standards. Equal amounts of total lipids (10 µg) were used in all analyses (34).

### RNA isolation, cDNA microarray, probe preparation, and hybridization

Total RNA was isolated by homogenization of 50-100 mg piece of frozen rat liver by using a polytrone PT-2000 (Kinematica AG, Malters, Switzerland) followed by the phenol-chloroform protocol supplied by the TRI Reagent-based method (Sigma-Aldrich). All samples were treated with RNAse-free DNAse set (Promega, Madison, WI, USA) and RNA was further purified by using the RNeasy Micro Kit (Qiagen, Valencia, CA, USA) following manufacturer’s recommendations. RNA yields were measured by UV absorbance and the quality of total RNA was analyzed by using a 2100 Bioanalyzer (Agilent, Palo Alto, CA, USA). A microarray containing 27000 rat 70-mer oligo probe sets produced at the KTH Microarray Center (www.biotech.kth.se) was used to evaluate the effects of hypothyroidism (TX), orchidectomy on TX (TXOX) and hormonal replacement in TXOX animals on liver transcriptome. Five μg of high-quality total RNA from the liver were reverse-transcribed and labelled with cyanine 3 (Cy3) and -5 (Cy5) using the ChipShot™ Direct Labeling System (Promega). After RNAse treatment, labelled cDNA was purified utilizing the QIAquick PCR Purification Kit (Qiagen). Hybridization was performed at 42°C for 16h and then, slides were washed following the Pronto™ Plus System protocol (Promega). Finally, slides were scanned using GenePix Scanner (Axon Instruments, Union City, CA, USA). To correct for the biological variability, 4 independent hybridizations were performed comparing individual animals from the different experimental groups for a total of 4 analyses (35).

### Microarray data processing and analysis

Image analysis was performed using the GenePix Pro 6.0 software as previously described (33). The LOWESS method was used to normalize the raw intensity data (36). If the measured probe sets were not present in at least 3 of the 4 chips, they were assumed to contain no information and therefore were eliminated to reduce data complexity. Differentially expressed genes (DEGs) were identified by using the Significance Analysis of Microarrays (SAM) statistical technique (37). A *q* value was assigned for each detectable gene in the array. This value is similar to a *p*-value, measuring the lowest false discovery rate (FDR < 0.05) at which differential expression of a gene is considered significant. In addition, a mean ratio of log2 > |0.32| value was chosen to describe up- or down-regulated genes. A completed list of DEGs is available as supplementary files and they have been deposited in Gene Expression Omnibus (GSE111718; www.ncbi.nlm.nih.gov/geo) (38).

### Functional- and pathway-enrichment analyses of DEGs

The Database for Annotation, Visualization and Integrated Discovery (DAVID; version 6.8), a widely used web-based tool for functional and pathway enrichment analyses (39), was used to perform Gene Ontology (GO) set enrichment analysis (GSEA) and Kyoto Encyclopedia of Genes and Genomes (KEGG) pathway enrichment analysis of DEGs. The GO terms and KEGG pathways with p<0.05 and a corrected *q* value (Benjamini) < 0.1 were selected.

### Protein-protein interactions network

The biological database and web tool for the retrieval of interacting genes STRING (40) was used to identify known and predicted protein-protein interactions and make a network of the overlapping DEGs. The results were visualized using Cytoscape software (41).

### Gene expression analysis by real-time quantitative-PCR

Briefly, 2 µg of total RNA were treated with RNase-free DNase I (Promega) and reverse transcribed using the iScript kit (Bio-Rad, Irvine, CA, USA) according to the manufacturer`s instructions. Two µl of cDNA served as a template in a 20 µl qPCR reaction mix containing the primers and SYBR Green PCR Master Mix (Diagenode, Seraing, Belgium). Quantification of gene expression was performed using the ABI PRISM^®^ 7000 SD RT-PCR System (Applied Biosystems, Thermo Fisher Scientific, Waltham, MA, USA). A relative standard curve was constructed with serial dilutions (1:1, 1:10, 1:100) using a pool of the cDNA generated from all animals used in the study. The amplification program consisted of 1 cycle of 95°C for 10 min, followed by 45 cycles of 95°C for 15 sec, annealing for 10 sec, and 72°C for 30 sec. The fluorescent intensity was measured at a specific acquisition temperature for each gene. Data were extracted and amplification plots generated with ABI SDS software (Applied Biosystems, Thermo Fisher Scientific). All amplifications were performed in duplicate, and C_t_ scores were averaged for subsequent calculations of relative expression values. The level of individual mRNA was normalized to the level of the housekeeping gene cyclophilin by using the Pfaffl method (42). Exon-specific primers (**Table S1**) were designed by the Primer 3 program (43).

### Statistical analysis

The significance of differences between groups was tested by one-way ANOVA, followed by *post hoc* comparisons of group means according to the GraphPad Prism 5 program. Statistical significance was reported if *P*<0.05 was achieved. For graphing purposes in the qPCR analysis, the relative expression levels were scaled so that the expression level of the INTACT group equaled one. Lipids classes and main FAs were additionally submitted to factor analysis by means of Principal Component Analysis (44). Factor scores were then analysed by two-way ANOVA to evaluate the combined effects of both hormonal treatments and their interactions. Multivariate analyses were performed using the SPSS package (version 15.0, SPSS Inc, Chicago, IL, USA).

## Results

### Influence of testosterone on somatotropic-liver axis in hypothyroid-hypogonadal male rats

**Figure 1:**
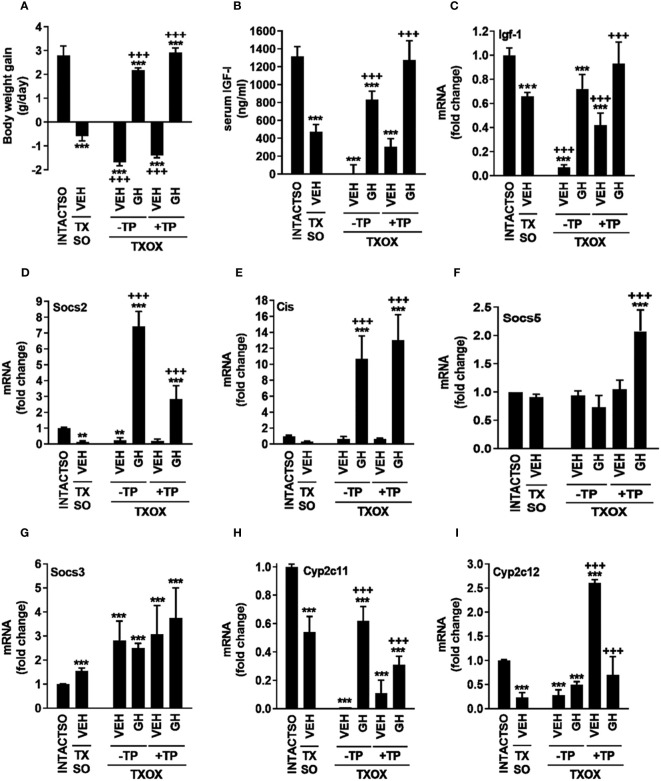
Influence of testosterone propionate (TP) on somatotropic-liver axis in hypothyroid male rats. Euthyroid testis-intact controls (INTACTSO group), hypothyroid testis-intact (TXSO group) and hypothyroid-orchiectomized (TXOX group) rat models were obtained as described in Material and Methods. TXSO group was treated with vehicle (VEH) and TXOX rats were administered with VEH (-TP) or TP (+TP) plus GH or VEH as described in Material and Methods. On postnatal day 94 (PND94), body weight gain **(A)**, circulating IGF-I **(B)**, and the hepatic mRNA expression levels of Igf-1 **(C)**, Socs2 **(D)**, Cis **(E)**, Socs5 **(F)**, Socs3 **(G)**, Cyp2c11 **(H)**, and Cyp2c12 **(I)** were determined as described in Material and Methods. Results are expressed as mean ± S.D (n=6). ^**^ *P*<0.01 versus VEH-treated INTACTSO group; ^***^ *P*<0.001 versus VEH-treated INTACTSO group; ^+++^ *P*<0.001 versus VEH-treated TXSO or TXOX groups, respectively.

Upon sacrifice on PND94, all MMI-treated groups showed lower or undetectable circulating T3 (ng/dl) [36.17 ± 5.43 (INTACTSO); 16.16 ± 2.73 (TXSO); 7.92 ± 4.84 (TXOX); 0,00 ± 0.00 (TXOXGH); 8.88 ± 3.07 (TXOXTP); 9.48 ± 0.96 (TXOXTPGH)] and T4 (ng/dl) [(1.90 ± 0.17 (INTACTSO) vs. 0)]. However, circulating levels of total T (ng/ml) in TXSO rats (10,14 ± 2,90) did not show differences compared to INTACTSO (10,66 ± 2,77) controls. Therefore, castration in TX rats was performed to explore the effects of TP replacement in the TXOX group. Consistently, 11-fold reduction of circulating T (ng/ml) (0,91 ± 0,12) was observed in the TXOX group when compared to non-castrated TXSO rats, whereas TP replacement led to 4-fold increase of T (4,10 ± 0,15) in the TXOXTP group. In contrast, circulating E2 (pg/ml) in TXOXTP rats (18,83 ± 8.45) was not significantly increased when compared to TXOX (12,50 ± 7,66). Next, as a first approach to assess the efficacy of TP replacement on somatotropic-liver axis, male phenotypic and functional biomarkers of GHR-STAT5b signaling were measured. Castration of TX rats (i.e., TXOX group) reduced body weight gain (**Figure 1A**), serum IGF-I (**Figure 1B**) and hepatic Igf-1 mRNA levels (**Figure 1C**) to a greater extent than non-orchiectomized but hypothyroid rats (i.e., TXSO). These changes were partially restored upon hormonal replacement with TP or pulsatile GH administration (**Figures 1A–C**). Interestingly, body weight gain and serum IGF-I were further increased when TP replacement was performed in the presence of GH (**Figures 1A–C**) which supports a positive cooperative interaction between T and GH on the somatotropic-liver axis.

To validate this cooperative interaction, in addition to hepatic Igf-1 mRNA levels (**Figure 1C**), several functional biomarkers of GHR-STAT5b signaling in the liver were also explored (e.g., Socs2, Cis, Cyp2c11, Cyp2c12). Castration of TX male rats (i.e., TXOX group) caused 80% decrease of Socs2 mRNA levels (**Figure 1D**). However, unlike TP, pulsed GH replacement induced Socs2 (**Figure 1D**) and Cis mRNA expression (**Figure 1E**). Furthermore, TP replacement partially prevented the positive effects of GH on Socs2 mRNA expression (**Figure 1D**), whereas GH-induced Cis mRNA levels remained unaffected (**Figure 1E**). Remarkably, TP and GH positively cooperated to regulate Socs5 gene expression (**Figure 1F**) but, conversely, Socs3 mRNA expression was increased in TXSO and TXOX (**Figure 1G**). As expected, the hepatic mRNA expression levels of gender differentiated genes were affected by TP or pulsatile GH replacement. First, the male specific Cyp2c11 gene was downregulated by 50% in TXSO rats, and it was abolished after castration (i.e., TXOX) (**Figure 1H**). Pulsed GH administration restored Cyp2c11 mRNA levels whereas the effect of TP replacement alone was lower. However, the efficacy of GH to induce Cyp2c11 mRNA was reduced in the presence of TP (**Figure 1H** **).** TP (**Table S2**), pulsed GH (**Table S3**) or combinatory treatment (**Table S4**) also increased expression of male-differentiated genes such as α-2u-globulin and major urinary proteins in TXOX rat liver. Paradoxically, TP replacement augmented the female-specific Cyp2c12 gene to higher level than age-matched intact and TXOX-vehicle treated rats (**Figure 1I**).

### Influence of testosterone on liver transcriptome in hypothyroid-castrated male rats

**Figure 2:**
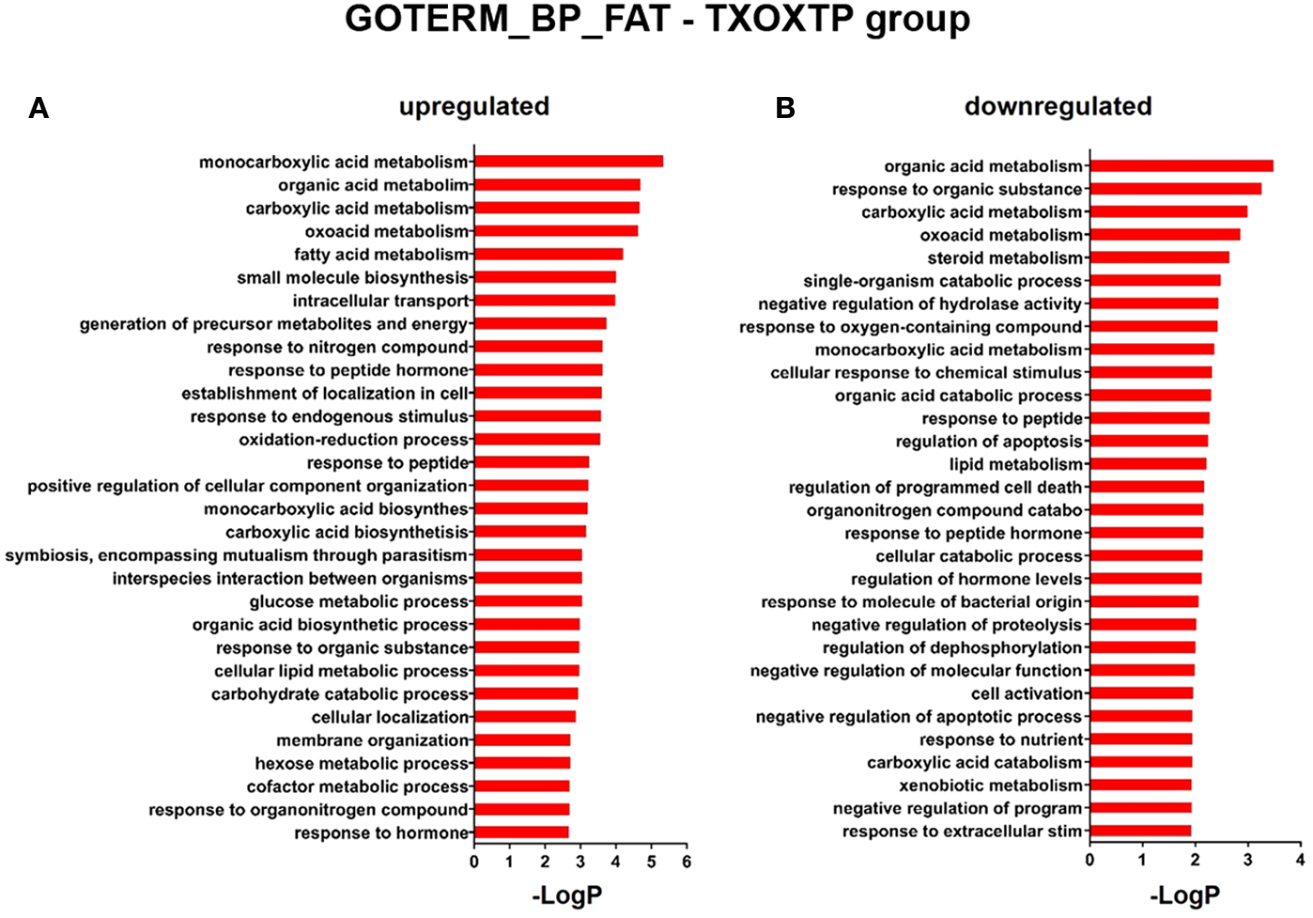
Functional enrichment analyses of differently expressed genes (DEGs) in testosterone propionate (TP)-treated hypothyroid-orchiectomized rats. Hypothyroid-orchiectomized (TXOX) rat model was obtained and treated with TP (TXOXTP group) as described in Material and Methods. DEGs in the liver were identified by DNA microarrays. This analysis was based on the SAM statistical technique and DEGs were discovered using a FDR < 5% and a mean ratio of log2 > |0.32|. The web-based tool for functional and pathway enrichment analyses (DAVID) was used to perform Gene Ontology (GO) set enrichment analysis (GSEA) of DEGs and to identify the Biological Process (BP) affected by TP. Top 30 up-regulated **(A)** and down-regulated **(B)** BPs enriched among the genes regulated in the TXOXTP liver. The GO terms with *P*<0.05 and a corrected *P* value [Benjamini **(B)**] < 0.1 were selected.

First, we performed a genome-wide analysis of gene expression to better understand the effects of T on liver transcriptome in TXOX rats. This analysis identified 611 DEGs (q<5%; Log_2_>|0.32|) that were distinctly regulated by TP (**Table S2**). Next, we identified genes involved in active biological processes (BPs) in the DEGs list by using GO functional clustering (39). GSEA of the 338 genes upregulated by TP (**Figure 2A**) revealed a significant over-representation of genes implicated in monocarboxylic acids metabolism, FA metabolism, biosynthesis of small molecules, generation of precursor metabolites and energy, response to nitrogen compounds, and glucose metabolism, among others. These BPs were also significantly upregulated by TP replacement in TXOX liver. In addition, TP administration to TXOX rats downregulated genes involved in the metabolism of organic and carboxylic acids, steroid metabolism, regulation of apoptosis, organic acid catabolism or lipid metabolism (**Figure 2B**). Accordingly, TP regulated several KEGG) intracellular signaling pathways linked to, among others, steroid metabolism (e.g., Cyp2c22; Cyp2d2; Ugt2b35; Hsd17b2), tricarboxylic acid cycle (TCA) cycle (e.g., Acly; Mdh1, Idh3B; Pck1), glycolysis/gluconeogenesis (e.g., Akr1a1; Gapdh; Pklr; Pck1), or chemical carcinogenesis (e.g., Cyp2c22; Ephx1; Cyp2c6) (**Table 1**). Overall, these results revealed an extensive re-programming of hepatic transcriptome by TP replacement in TXOX rats, particularly of those genes that play a functional role in glucose and lipid metabolism.

**Table 1:** Cellular signaling pathways regulated by testosterone propionate (TP) replacement in hypothyroid-orchiectomized rat liver.

Pathway name	up-regulated genes	down-regulated genes	*P*	B
Metabolic pathways	Acly; Akr1a1; Ndufb8; Baat; Chdh; Cyp2c22; Cox8a; Dgat2; Gapdh; Khk; Ldha; Mdh1; Prodh1; Pklr	Hibadh; ATP5Fl; Ndufb3; St6gal1; Ugt2b35; Ugdh; Aldh1a1; Aox1; Amacr; Asl; Cdo1; Cyp2c6; Cyp3a18; Dpys; H6pd; Hal; Hsd17b2; Idh3B; Otc; Pon3; Pck1; Pafah1b1; Sec1; Sqle; Tdo2	6.00E-04	1.10E-01
Biosynthesis of antibiotics	Acly; Akr1a1; Gapdh; Ldha; Mdh1; Prodh1; Pklr	Asl; Idh3B; Otc; Pck1; Sqle	1.23E-03	1.14E-01
Steroid hormone biosynthesis	Cyp2c22; Cyp2d2	Ugt2b35; Cyp2c6; Cyp3a18; Hsd17b2	1.21E-02	5.51E-01
Retinol metabolism	Cyp2c22	Ugt2b35; Aldh1a1; Aox1; Cyp2c6; Cyp3a18	1.34E-02	4.85E-01
Tricarboxylic acid (TCA) cycle	Acly; Mdh1	Idh3B; Pck1	1.67E-02	4.85E-01
Chemical carcinogenesis	Cyp2c22; Ephx1	Ugt2b35; Cyp2c6; Cyp3a18; Gstp1	1.93E-02	4.72E-01
Pyruvate metabolism	Ldha; Mdh1; Pklr	Pck1	3.02E-02	5.78E-01
Glycolysis/Gluconeogenesis	Akr1a1; Gapdh; Ldha; Pklr	Pck1	3.11E-02	5.41E-01
Ribosome	Rpl27a; Rpl28; Rpl4; Rps12; Rps15	Rpl17; Rps15a	7.31E-02	7.43E-01

Hypothyroid-orchiectomized rats (TXOX) were treated with vehicle (TXOX group) or TP (TXOXTP group) as described in Material and Methods. Differently expressed genes (DEGs) in the livers were identified by DNA microarrays. This analysis was based on the SAM statistical technique and DEGs were discovered using a FDR < 5% and a mean ratio of log2 > |0.32|. The web-based tool for functional and pathway enrichment analyses (DAVID) was used to identify the hepatic Kyoto Encyclopedia of Genes and Genomes (KEGG) pathways that were affected by TP. Table shows the pathway name (KEGG), up- and down-regulated genes, *P* value, and corrected *P* value [Benjamini (B)]. Gene abbreviations are shown in the supplementary legend to **Table 1**.

### Testosterone influences GH-regulated liver transcriptome in hypothyroid male rats

**Figure 3:**
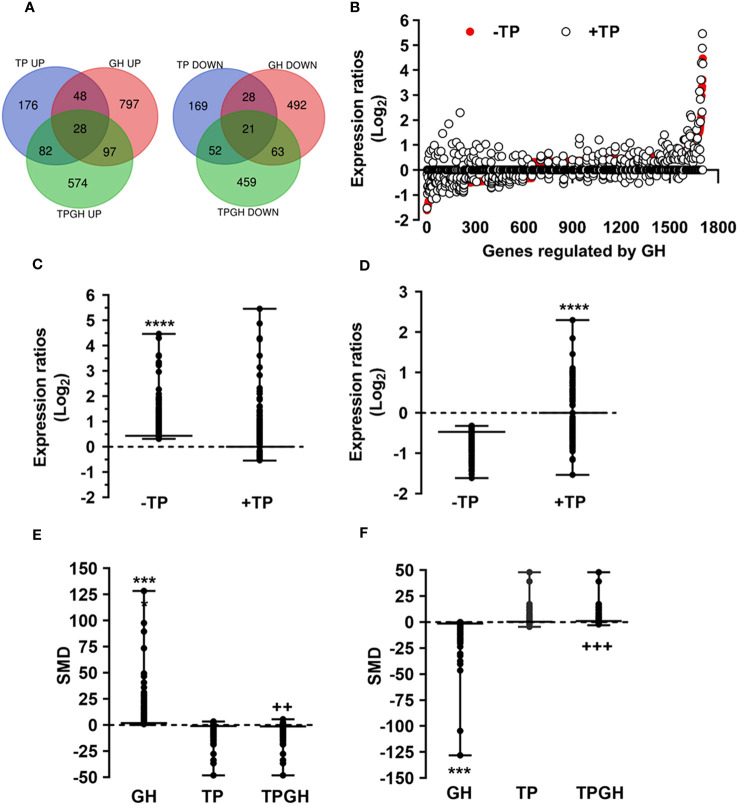
Testosterone propionate (TP) influences the gene expression profiling regulated by pulsed GH in hypothyroid-orchiectomized rat liver. Hypothyroid-orchiectomized (TXOX) rat model was obtained and treated with vehicle (-TP; TXOX group) or TP (+TP; TXOXTP group) plus GH (TXOXTPGH and TXOXGH groups) as described in Material and Methods. Differently expressed genes (DEGs) in the liver were identified by DNA microarrays. **(A)** Number of up- and downregulated genes in the TXOXGH, TXOXTP or TXOXTPGH groups. The overlapping areas show genes which their expression was altered by GH in the TXOXGH and TXOXTPGH groups. **(B)** Genes regulated by GH in the TXOXGH and TXOXTPGH groups. The X axis of the graph shows the individual genes arranged according to their order value of decreased and increased expression. The Y axis of the graph shows the log2 ratio of the transcript signals regulated by GH. **(C, D)** Statistical evaluation (box plots) of the differences in the mean expression changes of genes induced **(C)** or repressed **(D)** by GH in the TXOXGH or TXOXTPGH groups. ^****^ *P*<0.0001 *versus* TXOXTPGH group. **(E, F)** SAM multiclass analysis that identifies genes whose mean expression values (SMD) were upregulated **(E)** or downregulated **(F)** by GH in TXOXGH, TXOXTP and TXOXTPGH groups. ^***^ *P*<0.001 versus TXOXTP or TXOXTPGH groups, respectively; ^++^ *P*<0.01 versus TXOXGH or TXOXTP groups, respectively; ^+++^ *P*<0.001 versus TXOXGH or TXOXTP groups, respectively.

**Figure 4:**
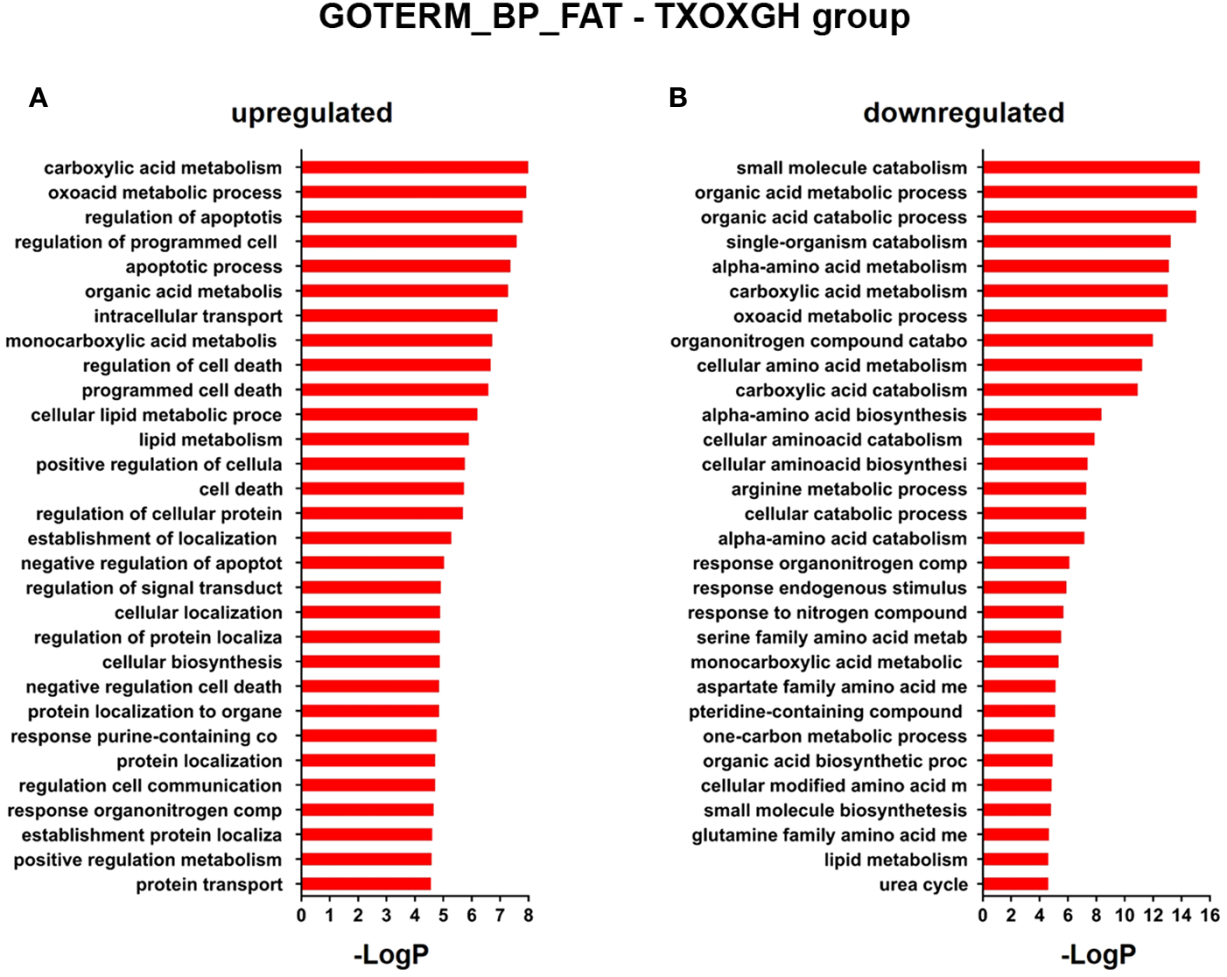
Functional enrichment analyses of differently expressed genes (DEGs) in GH-treated hypothyroid-orchiectomized rats. Hypothyroid-orchiectomized (TXOX) rat model was obtained and treated with GH (TXOXGH group) as described in Material and Methods. DEGs in the liver were identified by DNA microarrays. This analysis was based on the SAM statistical technique and DEGs were discovered using a FDR < 5% and a mean ratio of log2 > |0.32|. The web-based tool for functional and pathway enrichment analyses (DAVID) was used to perform Gene Ontology (GO) set enrichment analysis (GSEA) of DEGs and to identify the Biological Process (BP) affected by GH. Top 20 up-regulated **(A)** and down-regulated **(B)** BPs enriched among the genes regulated in the TXOXGH liver. The GO terms with *P*<0.05 and a corrected *P* value [Benjamini **(B)**] < 0.1 were selected.

**Figure 5:**
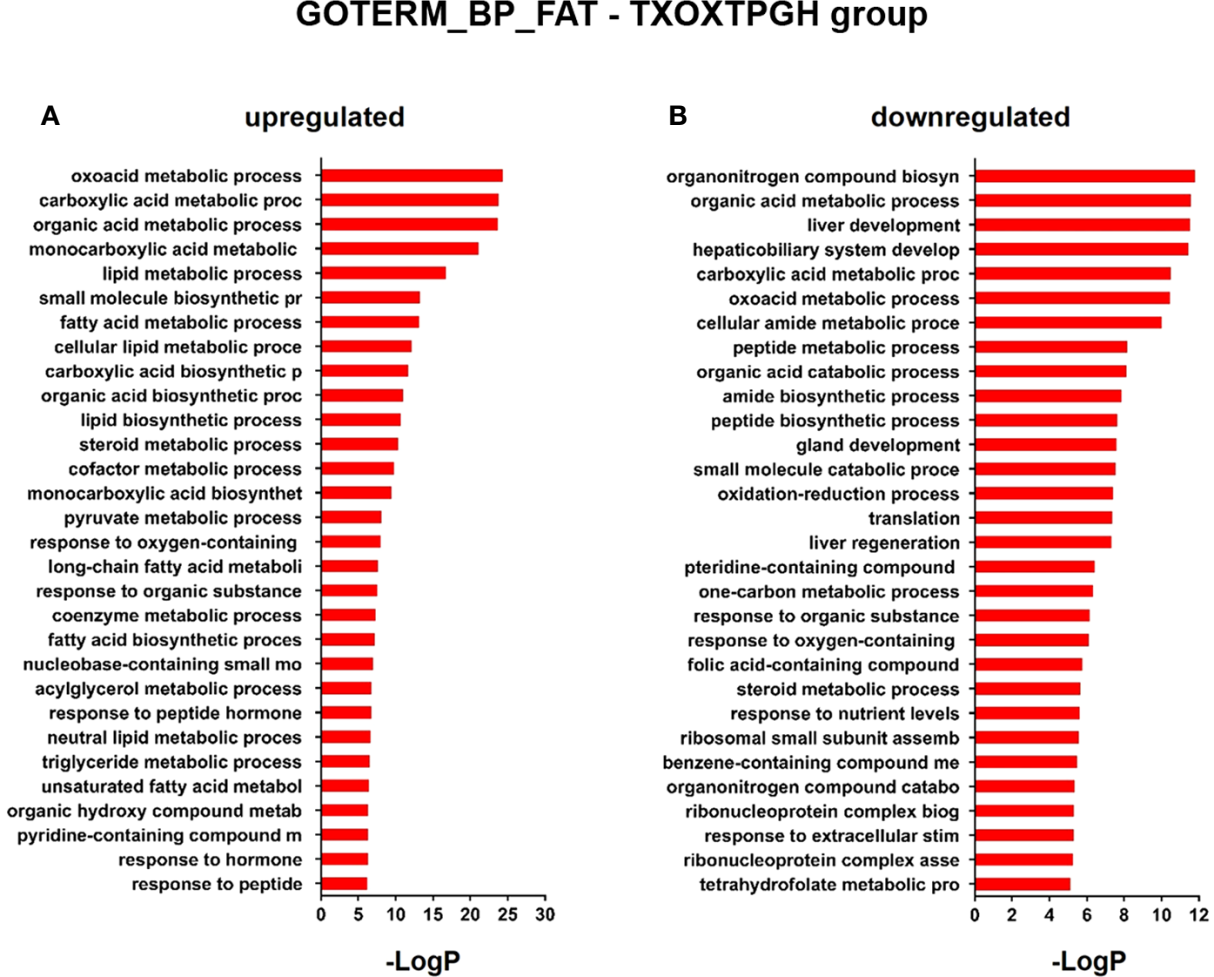
Functional enrichment analyses of differently expressed genes (DEGs) in testosterone propionate (TP) and GH-treated hypothyroid-orchiectomized rats. Hypothyroid-orchiectomized (TXOX) rat model was obtained and treated with TP plus GH (TXOXTPGH group) as described in Material and Methods. DEGs in the liver were identified by DNA microarrays. This analysis was based on the SAM statistical technique and DEGs were discovered using a FDR < 5% and a mean ratio of log2 > |0.32|. The web-based tool for functional and pathway enrichment analyses (DAVID) was used to perform Gene Ontology (GO) set enrichment analysis (GSEA) of DEGs and to identify the Biological Process (BP) affected by TP and GH. Top 30 up-regulated **(A)** and down-regulated **(B)** BPs enriched among the genes regulated in the TXOXTPGH liver. The GO terms with *P*<0.05 and a corrected *P* value [Benjamini **(B)**] < 0.1 were selected.

Since the liver is a primary organ in which T and GH interact to enhance body growth and composition (13), we next performed a genome-wide analysis of gene expression to better understand the influence of TP on GH-regulated hepatic transcriptome in TXOX rats. First, we identified DEGs (**Table S3**) and KEGG signaling pathways (**Table S4**) induced by pulsed GH administration to TXOX rats. Second, the similarities in DEGs induced by TP or GH were analysed, and 125 significantly regulated transcripts (FDR<5%; q<5%; log_2_>|0.32|) were identified in both TXOXTP and TXOXGH groups (**Figure 3A**). A Spearman rank correlation test comparing the effects of TP or GH on these DEGs yielded a strong positive correlation (r=0.6016; *P*<0.0001) between the effects of these hormones on liver transcriptome. Accordingly, 90% of these genes were regulated in the same way by TP and GH. Third, the transcription profiles of TXOX rats simultaneously treated with TP and GH were analyzed. A comparative analysis of DEGs in TXOXGH (**Table S3**) or TXOXTPGH (**Table S5**) groups revealed that T influenced a large fraction of GH-regulated genes (**Figures 3B–D**). Accordingly, in the absence of TP, the average expression changes (Log_2_) across four independent hybridizations were 0.54 ± 0.01 and -0.52 ± 0.01 for GH-induced (**Figure 3C**) and GH-repressed (**Figure 3D**) genes, respectively, whereas the average fold regulation for the same set of DEGs in the presence of TP was 0.11 ± 0.01 and -0.05 ± 0.01, respectively. These significant differences (*P*<0.0001) showed that TP replacement inhibited several GH-regulated genes in the liver of TXOX rats. Finally, SAM multiclass analysis (37) (**Figures 3E, F** and **Table S6**) identified genes that were regulated by GH whose mean expression values (SMD) were significantly different from those in TP- or TPGH-treated TXOX rats. DEGs by GH replacement in the presence of TP (i.e., TXOXTPGH) (**Table S5**) were functionally linked to KEGG signaling pathways associated to TCA cycle, PPAR, chemical carcinogenesis, steroid hormone biosynthesis, FA metabolism, amino acids metabolism, glycolysis/gluconeogenesis and diseases associated to glucose and lipid metabolism (e.g., NAFLD) (**Table 2**). Similarly, GH replacement whose SMD were significantly different from those in TP- or TPGH-treated TXOX rats (**Table S6**) were functionally linked to KEGG pathways associated to ribosome regulation, TCA cycle, oxidative phosphorylation, FoxO (e.g., Igf1, Tgfbr1, Homer3, Kras), FA degradation (e.g., Gcdh, Echs1, Ehhadh), glycolysis/gluconeogenesis, arginine biosynthesis (e.g. Sds, Mat1a, Pah, Otc, Cps1, Cth), and to diseases such as prostate cancer and NAFLD. Extensive similarity was also detected between BPs that were upregulated by pulsed GH (**Figure 4**) when compared with those regulated by GH in the presence of TP (i.e., TXOXTPGH) (**Figure 5**).

**Table 2:** Influence of testosterone propionate (TP) replacement on cellular pathways (KEGG) regulated by pulsed growth hormone (GH) administration in hypothyroid rat liver.

Pathway name	up-regulated genes	down-regulated genes	*P*	B
Metabolic pathways	77	44	3.94E-16	1.14E-13
Tricarboxylic acid (TCA) cycle	Acly; Mdh1; Mdh2; Pck1; Pc; Pdhb; Sdhc; Sucla2	Aco1; Fh; Suclg2	9.63E-07	6.16E-05
PPAR signaling pathway	Acox3; Acsl4; Cpt2; Fabp1; Fads2; Ppard; Pck1; Rxra; Scd3; Slc27a5; Scp2	Acadm; Acox1; Cyp8b1; Ehhadh	1.67E-06	7.12E-05
Chemical carcinogenesis	Cyp2b2; Cyp2c22; Cyp2c24; Cyp2c6v1; Cyp2c7; Gsta5; Gsto1; Kyat1; Mgst1; Ugt1a6	Cyp2c23; Cyp2e1; Cyp3a18; Gstm2; Gstp1; Hsd11b1; Ugt2b1	3.13E-06	1.14E-04
Steroid hormone biosynthesis	Comt; Cyp2b2; Cyp2c22; Cyp2c24; Cyp2c6v1; Cyp2c7; Cyp2d2; Srd5a1; Ugt1a6	Cyp11a1; Cyp2c23; Cyp2e1; Cyp3a18; Hsd17b2; Hsd11b1; Ugt2b1	3.26E-06	1.04E-04
Ribosome	Mrpl34	LOC100911372; Mrpl32; Rpl13; Rpl14; Rpl17; Rpl19; Rpl22; Rpl23; Rpl32; Rpl34; Rpl37a; Rpl7; Rps14; Rps15a; Rps16; Rps17; Rps20; Rps24; Rps25; Rps27	1.15E-05	3.26E-04
Fatty acid metabolism	Acadv; Acox3; Acsl4; Cpt2; Elovl5; Fads1; Fads2; Scd3(LOC681458); Scd	Acadm; Acox1; Ehhadh	2.66E-05	6.81E-04
Biosynthesis of amino acids	Aldob; Ass1; Eno1; Got2; Gapdh; Pc; Pklr; Pkm; Shmt2; Tpi1	Aco1; Cth; Otc	2.95E-04	5.80E-03
Glycine, serine and threonine metabolism	Agxt; Bhmt; Chdh; Grhpr; Shmt2	Agxt2; Cth; Dmgdh; Pipox	3.23E-04	5.88E-03
Parkinson’s disease	Atp5d; Atp5a1; Atp5g3; Cyc1; Ndufv1; Atp5b; Cox6c; Ndufa4; Atp5g1; Ndufa10; Cox8a; Cox4i1; Sdhc; Cycs	Gnai3; Ndufb10; Pink1; Vdac2	5.78E-04	9.81E-03
Glycolysis/Gluconeogenesis	Aldh9a1; Akr1a1; Aldob; Eno1; Gapdh; Ldha; Pck1; Pdhb; Pklr; Pkm; Tpi1		1.25E-03	1.98E-02
Retinol metabolism	Cyp2b2; Cyp2c22; Cyp2c24; Cyp2c6v1; Cyp2c7; Retsat; Rpe65; Ugt1a6	Aldh1a1; Cyp2c23; Cyp3a18; Ugt2b1	1.36E-03	2.03E-02
Complement and coagulation cascades	F12; C8a; C8b; Fga; Fgg	F11; F13b; C1qb; C1s; C4bpb; Klkb1	1.55E-03	2.07E-02
Alzheimer’s disease	Apoe; Atp5a1; Atp5b; Atp5d; Atp5g1; Atp5g3; Cox4i1; Cox6c; Cox8a; Cycs; Cyc1; Gapdh; Lrp1; Mapk1; Ndufa10; Ndufv1; Ndufa4; Sdhc	Ndufb10	1.70E-03	2.06E-02
Linoleic acid metabolism	Cyp2c22; Cyp2c24; Cyp2c6v1; Cyp2c7	Cyp2c23; Cyp2e1; Cyp3a18; Pla2g16	2.37E-03	2.50E-02
Non-alcoholic fatty liver disease (NAFLD)	Adipor2; Cebpa; Cox4i1; Cox6c; Cox8a; Cycs; Cyc1; Ndufa10; Ndufv1; Ndufa4; Pklr; Rxra; Sdhc	Atf4; Cdc42; Cyp2e1; Ndufb10	3.07E-03	3.10E-02
Metabolism of xenobiotics by cytochrome P450	Akr7a3; Gsta5; Gsto1; Mgst1; Ugt1a6	Cyp2e1; Gstp1; Hsd11b1; Ugt2b1	4.49E-03	4.33E-02
Drug metabolism - cytochrome P450	Fmo9; Gsta5; Gsto1; Mgst1; Ugt1a6	Cyp2e1; Fmo5; Gstm2; Gstp1; Ugt2b1	4.94E-03	4.58E-02
Fatty acid degradation	Acadvl; Acox3; Acsl4; Aldh9a1; Cpt2	Acadm; Acox1; Ehhadh	5.23E-03	4.68E-02
Oxidative phosphorylation	Atp5a1; Atp5b; Atp5d; Atp5g1; Atp5g3; Cox4i1; Cox6c; Cox8a; Cyc1; Ndufa10; Ndufv1; Ndufa4; Sdhc	Ndufb10; Ppa2	6.27E-03	5.40E-02
Huntington’s disease	Atp5a1; Atp5b; Atp5d; Atp5g1; Atp5g3; Cox4i1; Cox6c; Cox8a; Cycs; Cyc1; Ndufa10; Ndufv1; Ndufa4; Rcor1; Sdhc	Ndufb10; Vdac2	2.43E-02	1.84E-01
Protein export	Sec61a1; Sec61a2; Srp19; Srpra	Sec62	2.94E-02	2.12E-01
Arachidonic acid metabolism	Cyp2b2; Cyp2c22; Cyp2c24; Cyp2c6v1; Cyp2c7; Cyp4f1	Cyp2c23; Cyp2e1; Pla2g16	3.19E-02	2.22E-01

Hypothyroid-orchiectomized rats (TXOX) were treated with vehicle (TXOX group) or testosterone propionate (TXOXTP) plus GH (TXOXTPGH group) as described in Material and Methods. Differently expressed genes (DEGs) in the liver were identified by DNA microarrays. This analysis was based on the SAM statistical technique and DEGs were discovered using a FDR < 5% and a mean ratio of log2 > |0.32|. The web-based tool for functional and pathway enrichment analyses (DAVID) was used to identify the Kyoto Encyclopedia of Genes and Genomes (KEGG) pathways that were affected by GH in the presence of TP. The table shows pathway name (KEGG), up- and down-regulated genes, *P* value, and corrected *P* value [Benjamini (B)]. Gene abbreviations are shown in the supplementary legend to **Table 2**.

**Figure 6:**
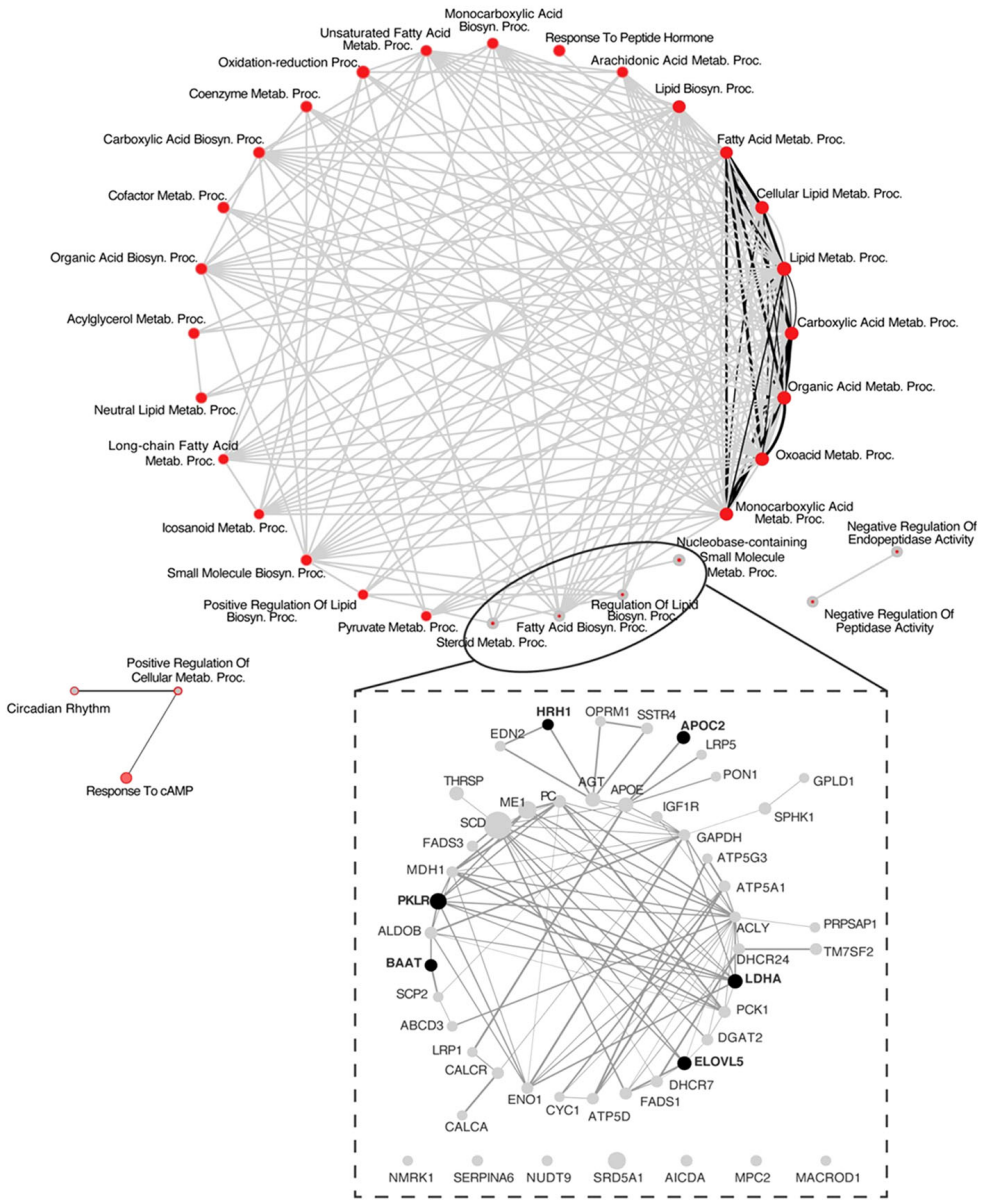
System biology network analysis of GH effects on liver transcriptome in testosterone propionate (TP)-treated hypothyroid-orchiectomized rats. Hypothyroid-orchiectomized (TXOX) rat model was obtained and treated with TP plus GH (TXOXTPGH group) as described in Material and Methods. Differently expressed genes (DEGs) in the liver were identified by DNA microarrays. The network presented in the top panel shows a comparative analysis of the biological processes (BPs) enriched among the genes upregulated in the TXOXTPGH group compared to those in the TXOXGH group obtained by using the web-based tool for functional and pathway enrichment analyses (DAVID) to perform Gene Ontology (GO) set enrichment analysis (GSEA). The network analysis was built using Cytoscape. On each node, inner circle corresponds to TP and GH treatment and the outer ring to GH treatment alone. On each node, red color represents significant enrichment of a given process and grey represents no significant enrichment. Connecting edges indicate shared genes between categories. Grey edges connect TPGH categories and black those of GH treatment alone. Genes corresponding to the four more enriched processes among genes upregulated by TPGH treatment are highlighted at the bottom of the top panel. The network presented in the lower panel shows protein-protein interaction network based on STRING database score of these highlighted genes, where the thickness of the edge is proportional to the STRING score. The size of each node is related to the fold change upregulation induced by TPGH administration compared to untreated TXOX rats. Finally, black nodes indicate genes also found upregulated by GH treatment alone.

**Figure 7:**
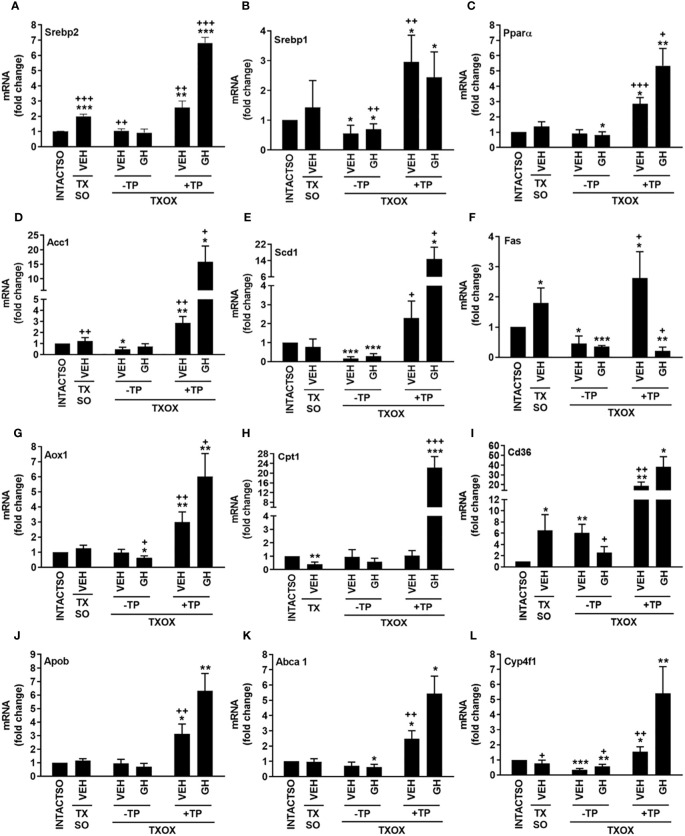
Influence of testosterone propionate (TP) on lipid genes in hypothyroid male rats. Euthyroid testis-intact controls (INTACTSO group), hypothyroid testis-intact (TXSO group) and hypothyroid-orchiectomized (TXOX group) rat models were obtained as described in Material and Methods. TXSO group was treated with vehicle (VEH) and TXOX rats were administered with VEH (-TP) or TP (+TP) plus GH or VEH as described in Material and Methods. On postnatal day 94 (PND94), the hepatic mRNA expression levels of Srebp2 **(A)**, Srebp1 **(B)**, Pparα **(C)**, Acc1 **(D)**, Scd1 **(E)**, Fas **(F)**, Aox1 **(G)**, Cpt1 **(H)**, Cd36 **(I)**, Apob **(J)** Abca1 **(K)** and Cyp4f1 **(L)** were determined by qRTPCR. Results are expressed as mean ± S.D. (n=6). ^*^ *P*<0.05; ^**^ *P*<0.01; ^***^ *P*<0.001 versus VEH-treated INTACTSO group; ^+^ *P*<0.05; ^++^ *P*<0.01; ^+++^ *P*<0.001 versus VEH-treated TXSO or TXOX groups.

The analysis of protein-protein interactions on TPGH (or GH)-regulated transcriptome in TXOX liver predicted interactions among proteins playing a crucial role in the regulation of biosynthesis of FA and steroid metabolism (**Figure 6**; labelled by black circle). However, several genes involved in FA biosynthesis were not modulated by pulsed GH but upregulated by TP (e.g., SCD, FASD, APOE, ME1, ENO1, DGAT2, PCK) (**Figure 6**; gray nodes). Interestingly, most of these genes were not upregulated by GH as a single treatment but were induced when GH was combined with TP (e.g., SRD5A1). This supports the hypothesis that the cooperation between T and GH is needed to induce the transcription of several genes and to stimulate the interaction between proteins (e.g., PKLR, BAAT, ELOVL5, APOC2, LDHA) (**Figure 6**; black nodes). TP also cooperates with GH to increase expression of genes involved in long chain FA (LCFA) synthesis (e.g., Acly) which suggests that this mechanism can promote LCFA synthesis. To validate the cooperative interaction between T and pulsed GH, several lipid biomarkers were explored by RTPCR (**Figure 7**). Castration of hypothyroid male rats (i.e., TXOX) reduced Srebp2, Srebp1, Acc1, Scd1, Fas or Cyp4f1, to a greater extent than non-orchiectomized but hypothyroid rats (i.e., TXSO). Interestingly, these changes were restored upon TP replacement and were further increased when TP was combined with pulsed GH which supports a positive cooperative interaction between these hormones to regulate lipid metabolism. This was not observed with the Fas gene which encodes the enzyme that catalyze *de novo* synthesis of FAs. In this case, the stimulative effect induced by TP on Fas gene was inhibited when TP was combined with GH (**Figure 7F**). Interestingly, only when TP was present, GH increased Srd5a1 gene expression (**Table S5**), which supports the hypothesis that an enhanced conversion of T to DHT could contribute to potentiate AR-dependent signaling in the liver. Overall, these results reveal an extensive overlapping and cooperation between T and pulsed GH in liver transcriptome linked to lipid metabolism.

### Testosterone influences hepatic lipid content in hypothyroid male rats

**Figure 8:**
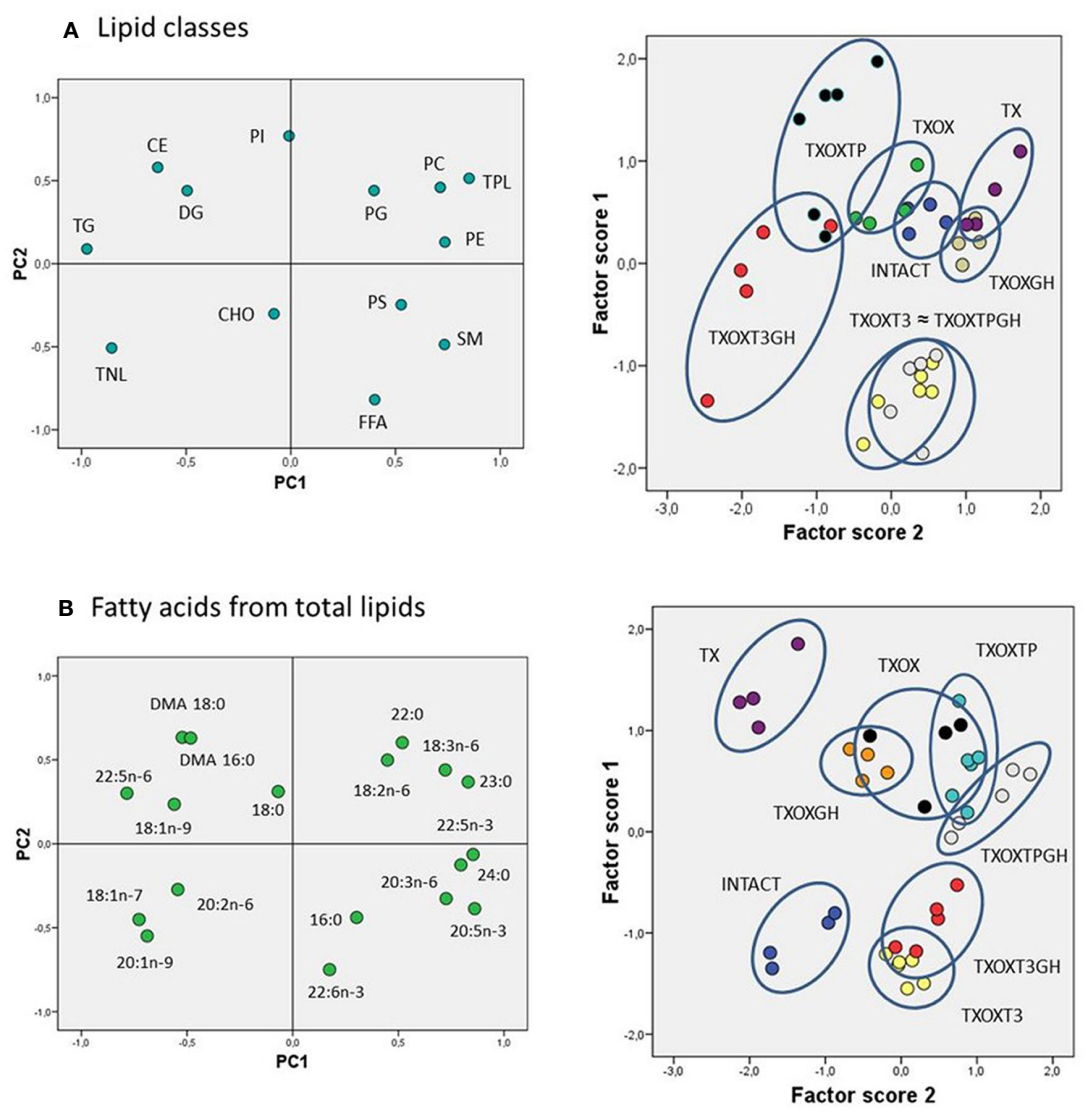
Lipidomic analysis in the liver of rat experimental models. Multivariate analyses (principal components (PC)) for liver lipid classes **(A)** and fatty acids from total lipids **(B)**. For both analyses, left panels represent the factor loadings of PC 1 (PC1) and 2 (PC2), and right panels the factor scores plot for each PC. Ellipses drawn in right panels (illustrating scatterplots for factors scores) indicate each hormonal cluster. CHO, total cholesterol; CE, cholesterol esters; DG, diacylglycerides; FFA, free fatty acids; PC, phosphatidylcholine; PE, phosphatidylethanolamine; PG, phosphatidylglycerol; PI, phosphatidylinositol; PS, phosphatidylserine; TG, triacylglycerols; TNL, total neutral lipids; TPL, total polar lipids; SM, sphingomyelin; DMA, dimethylacetals.

The transcriptome data suggested that T and GH cooperate to maintain lipid composition in the liver. Therefore, the effects of T on serum and hepatic lipid composition were first evaluated by quantitative analysis of lipid classes in TXOX rats (**Table 3**). The development of a hypothyroid state in male rats was accompanied by altered levels of circulating lipids (i.e., 2-fold increase of CHO and 3-fold decrease of TG). However, neither TP alone nor its combination with GH were able to normalize these variations. In contrast, TP replacement caused complex changes of hepatic lipids (**Table 3**). Given the complexity of these data, we initially performed Principal Component Analysis of lipid classes to discriminate experimental groups based on their lipid profiles (**Figure 8A**). The outcomes revealed that the two principal components explained 64% of overall variance. Principal Component 1 was negatively related to TG, cholesteryl esters and total neutral lipids; and positively to phosphatidylcholine, phosphatidylethanolamine, sphingomyelin, and total polar lipids. Principal Component 2 was mainly related to phosphatidylinositol and free FA (FFA) (**Figure 8A**, left panel). When we computed factor scores 1 and 2 from PC1 to pursue group simplification (**Figure 8A**, right panel), we obtained that INTACTSO group were represented by a discrete cluster with a small degree of intersection with TXOX, TXOXGH and TX groups. Best distinguishable groups were TXOXTP, TXOXT3GH, TXOXT3 and TXOXTPGH (these later two groups displaying nearly complete overlapping). Overall, it may be concluded that TP, GH and T3 differentially modify hepatic lipid metabolism, but their effects are clearly modulated by their combinations. This was particularly obvious for the interactions of the effects of TP or T3, on one side, and the presence of GH, on the other (**Figure 8A**, right panel).

**Table 3:** Effects of hormonal replacement on serum and hepatic lipid classes from hypothyroid rat liver.

Lipid classes	INTACTSO		TXOX		TXSO		TXOXGH		TXOXTP		TXOXTPGH	
	mean ± SEM		mean ± SEM		mean ± SEM		mean ± SEM		mean ± SEM		mean ± SEM	
Serum												
Glucose (g/dl)	141.17 ± 11.50		126.83 ± 15.33		133.50 ± 17.38		107.67 ± 17.33		115.17 ± 5.53		124.17 ± 8.13	
CHO (mg/dl)	67.17 ± 2.98	a	126.5 ± 10.11	bcd	130.17 ± 7.45	cd	129.33 ± 12.88	cd	138.00 ± 8.56	d	115.67 ± 4.25	bcd
TG (mg/dl)	133 ± 16.33	a	44 ± 5.09	a	56 ± 4.23	ab	43.83 ± 6.46	a	52.67 ± 4.34	a	64.67 ± 9.94	ab
Hepatic (%)												
LPC	0.00 ± 0.00		0.00 ± 0.00		0.00 ± 0.00		0.00 ± 0.00		0.00 ± 0.00		0.00 ± 0.00	
SM	0.86 ± 0.04	c	1.02 ± 0.05	cd	0.72 ± 0.11	bc	0.74 ± 0.17	bc	1.75 ± 0.13	e	0.44 ± 0.05	b
PC	25.28 ± 0.58	bc	26.95 ± 1.21	bcd	29.87 ± 0.84	d	28.48 ± 0.73	cd	30.65 ± 0.68	d	24.95 ± 0.75	b
PS	2.26 ± 0.04	ab	1.81 ± 0.2	a	3.48 ± 0.15	bc	2.75 ± 0.32	ab	4.96 ± 0.58	c	2.61 ± 0.19	ab
PI	11.11 ± 0.34	e	8.84 ± 0.33	de	9.47 ± 0.19	de	10.19 ± 0.26	e	4.16 ± 0.45	a	6.52 ± 0.24	bc
PG	5.53 ± 0.12	ab	5.28 ± 0.27	ab	6.03 ± 0.14	b	4.48 ± 0.53	a	5.16 ± 0.26	ab	5.37 ± 0.14	ab
PE	17.39 ± 0.3	b	18.08 ± 0.57	b	17.00 ± 0.57	b	17.63 ± 1.56	b	16.65 ± 1.10	b	12.30 ± 0.37	a
PLE	0.79 ± 0.21		0.45 ± 0.04		0.26 ± 0.02		0.30 ± 0.02		0.00 ± 0.00		0.00 ± 0.00	
DG	1.71 ± 0.26	b	0.92 ± 0.2	a	1.53 ± 0.16	ab	1.80 ± 0.06	b	1.38 ± 0.10	ab	2.14 ± 0.19	b
CHO	14.71 ± 0.68	abc	17.07 ± 0.28	d	13.45 ± 0.61	ab	13.18 ± 0.25	a	15.27 ± 0.30	abcd	15.70 ± 0.37	cd
FFA	7.94 ± 0.54	bc	7.15 ± 0.88	b	2.43 ± 0.44	a	2.48 ± 0.49	a	11.80 ± 1.20	c	1.21 ± 0.25	a
TG	8.35 ± 0.36	ab	7.84 ± 0.88	ab	8.48 ± 0.92	ab	11.46 ± 0.96	b	4.69 ± 0.90	a	20.66 ± 1.25	c
CE	4.08 ± 0.29	a	4.59 ± 0.1	ab	7.29 ± 0.58	c	6.50 ± 0.12	bc	3.53 ± 0.57	a	8.10 ± 0.70	c
TPL	63.21 ± 0.58	b	62.43 ± 1.31	b	66.82 ± 1.39	b	64.58 ± 0.67	b	63.33 ± 2.12	b	52.18 ± 1.24	a
TNL	36.79 ± 0.58	a	37.57 ± 1.31	b	33.18 ± 1.39	a	35.42 ± 0.67	a	36.67 ± 2.12	a	47.82 ± 1.24	b

Euthyroid testis-intact shame-operated (SO) controls (INTACTSO), hypothyroid SO (TXSO) and hypothyroid-orchiectomized (TXOX) rat models were obtained as described in Material and Methods. Administration of testosterone propionate (TXOXTP) or vehicle (VEH) to TXOX rats was performed for 20 days. Then, VEH (TX, TXOX), GH (TXOXGH), TP (TXOXTP) or TP plus GH (TXOXTPGH) replacements were administered for additional 7 days. On postnatal day 94 (PND94), animals were euthanized, and serum and hepatic lipid classes were measured. Data are expressed as mean ± SEM for 5 different animals. Values represent weight percent of total lipid. Values were submitted to ANOVA followed by post hoc Tukey’s test. Values in the same row with different lowercase letters are significantly different with *P*<0.05. Abbreviations for lipid classes are shown in the supplementary legend to **Table 3**.

To provide a quantitative assessment of hormonal interactions, we next performed two-way ANOVA on factor scores as well as on individual lipid classes. We found that there was a dependent relationship between all the hormones studied (particularly for GH), and interactions between them were identified for the first two components. For factor score 1, main dependency was obtained for GH (F=19.191, *P*=0.000). Further, a clear interaction was observed for TP and T3 (F=30.11, *P*=0001), and between GH and TP (F=19.32, *P*=0.000). For factor score 2, the main determinant of variance was the state of TP (F=8.36, *P*=0.007) followed by T3 (F=3.10, *P*=0.088). There were also significant interactions between T3 and TP (F=13.49, *P*=0.001) as well as between GH and T3 (F=24.69, *P*=0.000). Provided these multivariate results, we next compared individual lipid classes in the liver focused on the main parameters determining factor score 1. Thus, TXOX animals exhibited highest hepatic CHO content and decreased levels of TG and DG compared to the INTACTSO group. The highest levels of hepatic CHO were found in TXOX rats whereas the lowest values were observed in TXOXGH rats [**Table 3** and (33)]. In general, changes in cholesteryl esters levels were found to be negatively related to variations in CHO levels across groups (r=0.5901, *P*=0.0002). Cholesteryl esters were highly increased by GH treatment and reached lowest levels after TP (that together exhibited a certain degree of antagonism). Compared to INTACTSO rats, TG levels were not affected in vehicle-treated TXOX but were considerably increased by GH (TXOXGH) and reached minimal levels after TP (TXOXTP) treatment (**Table 3**).

The effects of TP were counteracted by the presence of GH, indicating an antagonistic effect of GH on T activity. In parallel, GH caused a dramatic reduction in hepatic levels of FFA, while TP induced the opposite effect. Noteworthy, changes in FFA negatively correlated with changes in DG (r=0.5296; *P*=0.0006). Interestingly, TP treatment of TXOX animals resulted in further FFA accumulation in the liver. The highest reduction of DG and increase of FFA was observed in TXOX rats treated with T3 plus GH (i.e., TXOXT3GH) (data not shown). However, the opposite response was observed in TXOX rats treated with TP plus GH (i.e., TXOXTPGH). These results indicate that, while GH effects are modulated by TP, the effects of this interaction are permissive. Furthermore, the combination of GH and TP significantly increased total neutral lipids due to changes in hepatic contents of CHO, cholesteryl esters, TG and DG. On the other hand, total polar lipids showed a reciprocal behavior to total neutral lipids. These data indicate a strong remodeling effect of hormonal treatments on hepatic phospholipids. Combination of TP and GH (but not GH alone) caused a significant reduction of total polar lipids, due to decreased phosphatidylcholine and phosphatidylethanolamine. Overall, these results suggest that hormonal replacement with GH, TP or TPGH can induce different hepatic lipid profiles in hypothyroid subjects. The main difference between GH and TP treatments was that TG levels were significantly increased by GH and reduced by TP. These data pinpoint to an anabolic effect leading to esterification of FFA into DG and TG triggered by GH, which was turned into catabolic by TP.

In order to better understand the influence of hormonal replacement on hepatic FA profiles (**Table 4**), we performed multivariate analyses of the whole dataset. Principal Component Analysis results showed that two Principal Components may explain 52% of total variance (**Figure 8B**, left panel). Principal Component 1 was negatively related to 22:5n6, 18:1n9, and 18:1n7, and positively to saturated FA (SFA) (23:0, 24:0), n-3 long chain polyunsaturated FA (LCPUFA) (22:5n3, 20:5n3), and n-6 LCPUFA (20:3n6). Principal Component 2 was mainly related to 20:4n6 (negatively) and 16:1n7 (positively). Computed factor scores 1 and 2 allowed the discrimination between different hormonal treatments into four major clusters. The INTACTSO and TXSO groups were represented by two discrete clusters whereas the T3 and TP groups appeared in differentiated clouds with certain degree of intersection (**Figure 8B**, right panel). Untreated TXOX group exhibited marginal intersections with TXOXGH and TXOXTP, but none with TP in the presence of GH (**Figure 8B**, right panel). Analyses of dependencies and interactions for the effects of different hormones on FA from total lipids were performed by multiple analyses of variance. The results for factor score 1 indicated that T3 was the main discriminating hormone (F=158.68, *P*=0.000), which also displayed some interaction with GH (F=3.47, *P*=0.072). However, for factor score 2, main dependences were observed for the three hormones individually (highly significant for T3: F=8.58, *P*=0.006), but only significant interactions with GH were detected for TP (F=8.87, *P*=0.006). Given these results, we next focused on main dependencies displayed by hormonal treatments on the composition of major FAs. Thus, compared to TXOX, TXOXTP animals displayed slightly lower levels of monoenoic FA (MUFA), while SFA and polyunsaturated FA (PUFA) remained unaffected (**Table 4**).

**Table 4:** Effects of hormonal replacement on fatty acid composition from total lipids in hypothyroid rat liver.

TOTAL LIPIDS	INTACTSO		TXOX		TXSO		TXOXGH		TXOXTP		TXOXTPGH	
	mean ± SEM		mean ± SEM		mean ± SEM		mean ± SEM		mean ± SEM		mean ± SEM	
Fatty acids												
C 14: 0	0.51 ± 0.06		0.58 ± 0.06		0.72 ± 0.09		0.49 ± 0.05		0.62 ± 0.05		0.48 ± 0.07	
C 15: 0	0.40 ± 0.03	cd	0.37 ± 0.01	bcd	0.46 ± 0.06	d	0.33 ± 0.02	abcd	0.37 ± 0.02	bcd	0.28 ± 0.01	abc
C 16: 0	19.81 ± 0.47	ab	20.38 ± 0.47	ab	22.09 ± 0.36	b	19.10 ± 0.26	a	19.91 ± 0.81	ab	19.61 ± 0.31	ab
C 16:1 n-9	0.30 ± 0.03	ab	0.44 ± 0.02	abc	0.57 ± 0.03	c	0.39 ± 0.03	abc	0.49 ± 0.04	bc	0.41 ± 0.03	abc
C 16: 1 n-7	2.16 ± 0.38		2.11 ± 0.65		2.85 ± 0.44		1.54 ± 0.27		1.65 ± 0.16		2.30 ± 0.21	
C 18: 0	13.17 ± 0.20	a	15.65 ± 0.33	bcd	13.97 ± 0.31	ab	14.70 ± 0.23	abc	16.39 ± 0.57	cd	12.62 ± 0.43	a
C 18: 1 n-9	10.61 ± 0.47	a	11.32 ± 1.07	a	10.50 ± 0.74	a	10.39 ± 0.49	a	10.17 ± 0.68	a	15.45 ± 0.54	b
C 18: 1 n-7	5.59 ± 0.38	c	2.71 ± 0.24	a	2.88 ± 0.12	a	2.99 ± 0.19	a	2.74 ± 0.14	a	3.05 ± 0.15	ab
C 18: 1 n-5	0.21 ± 0.03		0.10 ± 0.02		0.14 ± 0.01		0.11 ± 0.02		0.16 ± 0.01		0.13 ± 0.03	
C 18: 2 n-6	15.80 ± 0.91	a	17.45 ± 0.85	ab	15.87 ± 1.67	a	17.06 ± 0.94	ab	17.19 ± 0.48	ab	19.73 ± 0.51	b
C 18: 3 n-3	0.16 ± 0.03	a	0.74 ± 0.06	b	0.65 ± 0.07	b	0.62 ± 0.02	b	0.80 ± 0.08	b	1.48 ± 0.22	c
C 20: 2 n-6	0.56 ± 0.05	c	0.23 ± 0.03	ab	0.16 ± 0.01	a	0.31 ± 0.01	b	0.24 ± 0.01	ab	0.19 ± 0.02	a
C 20: 3 n-6	0.75 ± 0.04	bcd	1.36 ± 0.23	e	1.30 ± 0.14	e	0.77 ± 0.03	cd	1.11 ± 0.15	de	0.45 ± 0.05	abc
C 20: 4 n-6	19.00 ± 0.92	b	17.00 ± 1.15	ab	13.88 ± 0.38	a	20.17 ± 0.36	c	18.82 ± 0.63	b	14.56 ± 0.35	a
C 20: 5 n-3	0.17 ± 0.04	bc	0.41 ± 0.05	d	1.02 ± 0.15	e	0.30 ± 0.05	cd	0.27 ± 0.05	cd	0.28 ± 0.02	cd
C 22: 0	0.04 ± 0.03	a	0.17 ± 0.01	b	0.12 ± 0.01	b	0.17 ± 0.00	b	0.19 ± 0.02	b	0.16 ± 0.01	b
C 23: 0	0.08 ± 0.01	ab	0.17 ± 0.00	cd	0.15 ± 0.01	cd	0.16 ± 0.01	cd	0.20 ± 0.01	d	0.17 ± 0.02	cd
C 22: 4 n-6	0.76 ± 0.09	d	0.53 ± 0.02	c	0.23 ± 0.04	a	0.82 ± 0.07	d	0.32 ± 0.02	ab	0.43 ± 0.05	bc
C 22: 5 n-6	0.98 ± 0.23	bcd	0.53 ± 0.05	b	0.19 ± 0.06	a	0.77 ± 0.11	bc	0.71 ± 0.06	bc	0.93 ± 0.14	bcd
C 22: 5 n-3	0.77 ± 0.04	b	1.32 ± 0.13	c	1.30 ± 0.03	c	1.00 ± 0.09	bc	1.02 ± 0.09	bc	1.21 ± 0.07	c
C 24: 0	0.44 ± 0.04	abc	0.59 ± 0.02	bc	0.63 ± 0.06	c	0.59 ± 0.05	bc	0.58 ± 0.05	bc	0.41 ± 0.04	a
C 22: 6 n-3	4.39 ± 0.19	c	3.10 ± 0.38	ab	7.37 ± 0.20	d	4.43 ± 0.30	c	3.44 ± 0.14	abc	3.03 ± 0.11	a
C 24: 1 n-9	0.17 ± 0.01	ab	0.22 ± 0.01	bcd	0.30 ± 0.02	d	0.21 ± 0.01	abc	0.26 ± 0.03	cd	0.14 ± 0.01	a
Totals												
SFA	35.09 ± 0.33	ab	38.52 ± 0.79	c	38.71 ± 0.40	c	36.14 ± 0.36	abc	38.99 ± 1.03	c	34.28 ± 0.37	a
MUFA	19.66 ± 1.11	abc	17.34 ± 1.93	ab	17.81 ± 1.33	ab	16.06 ± 0.87	a	15.72 ± 0.84	a	21.80 ± 0.79	bc
PUFA	43.75 ± 1.51	ab	43.25 ± 2.01	ab	42.45 ± 1.56	ab	47.01 ± 0.90	b	44.43 ± 0.72	b	43.06 ± 0.59	ab
n-3/n-6	0.15 ± 0.01	a	0.14 ± 0.00	a	0.31 ± 0.02	b	0.15 ± 0.01	a	0.13 ± 0.00	a	0.13 ± 0.00	a
UI	171.36 ± 2.96	bc	160.74 ± 5.47	ab	170.35 ± 1.36	ab	177.95 ± 2.47	c	164.98 ± 2.95	ab	159.98 ± 2.02	ab
PI	125.47 ± 2.36	abc	114.39 ± 6.06	a	137.66 ± 0.73	c	132.75 ± 3.58	bc	119.55 ± 2.75	ab	109.61 ± 2.47	a
SFA/n-3	6.27 ± 0.18	bc	7.56 ± 0.57	bcd	3.89 ± 0.08	a	6.00 ± 0.31	b	7.76 ± 0.34	cd	6.81 ± 0.18	bcd
SFA/n-9	3.05 ± 0.10	b	3.19 ± 0.28	b	3.33 ± 0.22	b	3.21 ± 0.16	b	3.59 ± 0.27	b	2.12 ± 0.09	a

Euthyroid testis-intact shame-operated (SO) controls (INTACTSO), hypothyroid SO (TXSO) and hypothyroid-orchiectomized (TXOX) rat models were obtained as described in Material and Methods. Administration of testosterone propionate (TXOXTP) or vehicle (VEH) to TXOX rats was performed for 20 days. Then, VEH (TX, TXOX), GH (TXOXGH), TP (TXOXTP) or TP plus GH (TXOXTPGH) replacements were administered for additional 7 days. On postnatal day 94 (PND94), animals were euthanized, and fatty acids and total hepatic lipids were measured. Data are expressed as mean ± SEM for 5 different animals. Values represent weight percent of total lipid. Values were submitted to ANOVA followed by post hoc Tukey’s test. Values in the same row with different lowercase letters are significantly different with *P*<0.05. Abbreviations for total lipids are shown in the supplementary legend to **Table 4**.

Hypothyroidism resulted in the accumulation of SFA in the liver, which was maintained upon castration. Comparison of TXOX data with those of TXSO suggested that many of the effects on FA contents were due to deficiency of gonadal hormones. Further, in the presence of GH (i.e., TXOXTPGH), the most notable changes were those introduced by T (**Table 4**). GH treatment was able to restore SFA to the levels of INTACTSO animals, an effect further enhanced by co-treatment with T. However, the comparison of TXOX group with TXSO revealed a discrete effect of gonadal hormones on hepatic FAs. This apparent absence of T effects was significantly changed when combined with GH. In the presence of T, (i.e., TXOXTPGH group), SFA levels dropped, while MUFA increased. These effects could be explained by the significant reduction of stearic acid (18:0) and the elevation of oleic acid (18:1n-9). For MUFA group, and particularly for 18:1n-9, a permissive action of GH on the effects of TP was evident, since levels of this major FA were highest in TXOXTPGH group, and significantly higher than in INTACTSO, TXOX, TXSO, TXOXGH and TXOXTP groups. Within n-3 or n-6 PUFAs series, no significant changes were observed by TP or TP plus GH treatments. However, linoleic acid (18:2n-6) was slightly increased and arachidonic acid (20:4n-6) reduced (as well as its elongation/desaturation intermediates 20:2n-6 and 20:3n-6) in the TXOXTPGH group, indicating that GH decreased the biosynthesis of essential n-6 LCPUFA in the presence of T. Within the n-3 LCPUFA series, TP alone or combined with GH (i.e., TXOXTPGH group) failed to modify levels of docosahexaenoic acid (DHA; 22:6n-3) and eicosapentaenoic acid (EPA; 20:5n-3) when compared to TXOX group. In this sense, the interaction between T and GH is particularly illustrative since DHA levels increased when GH was administered alone (**Table 4**). In agreement, comparison of TXOX and TXOXTPGH groups with TXSO rats, suggested an important role of gonadal hormones on DHA and arachidonic acid levels since they were higher and lower in TXSO rats, respectively. This effect may be caused by orchiectomy. It might be concluded a main role of T plus GH in the configuration of hepatic FA profiles, but these effects are modulated by complex interactions between them.

### Androgenic nature of testosterone effects on liver transcriptome in hypothyroid male rats

**Figure 9:**
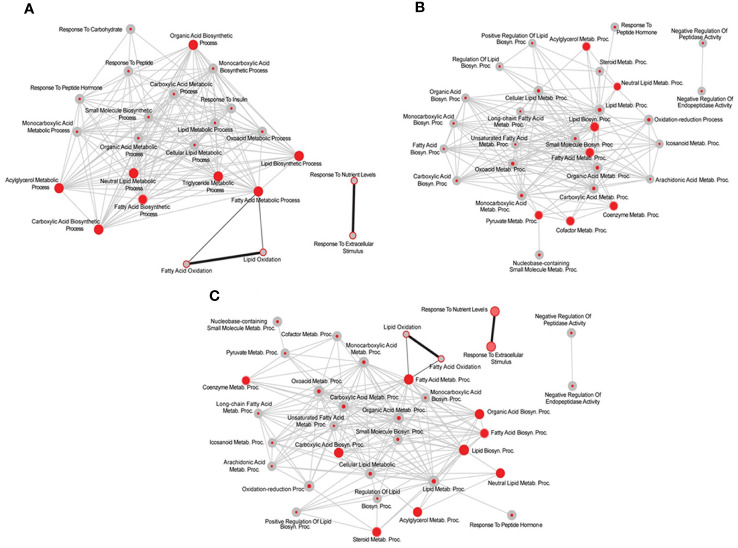
Limited overlap between testosterone propionate (TP) and estradiol (E2)-regulated liver transcriptome in hypothyroid-orchiectomized rats. Comparative analysis of the biological processes (BPs) enriched among the genes upregulated by TP, E2 and/or GH treatments in the liver of hypothyroid-orchiectomized (TXOX) rats. Networks were obtained by using the web-based tool for functional and pathway enrichment analyses (DAVID) to perform Gene Ontology (GO) set enrichment analysis (GSEA). TP-induced effects compared to those of E2 **(A)**; TP- and GH-induced effects compared to those of E2 and GH **(B)**; TP- and GH-induced effects compared to those of E2 **(C)**. On each node, inner circle represents TP- **(A)**; TP- and GH- induced effects **(B, C)**; and outer ring corresponds to E2- **(A, C)** or E2- and GH- induced effects **(B)**. On each comparison, GSEA was used to statistically evaluate the overlap between the conditions tested.

Finally, the potential androgenic, estrogenic or both effects of TP replacement on liver transcriptome and lipid composition were assessed. First, T-regulated transcriptome (**Table S2**) was compared to those genes upregulated by E2 (33) but no significant correlation between the DEG datasets was found, and GSEA comparison of the BPs upregulated by TP and E2 showed limited overlap (**Figure 9A**). E2 treatment induced expression of genes involved in FA oxidation (e.g., PPAR-dependent signaling) while T upregulated those genes linked to FA biosynthesis which agrees with an increased FFA accumulation in TXOXTP liver (**Table 4**). Second, TPGH-regulated transcriptome (**Table S3**) was compared to those genes upregulated by E2GH (33) but a limited (6% of the genes) and significant correlation (q<0.0001) was found (**Figure 9B** **).** Strikingly, the overlap between TPGH and E2 administered alone was 16% (q<0.0001) (**Figure 9C**). However, when TP- (or TPGH-) upregulated genes were compared to those modulated by E2 (or E2GH), there were global different effects, despite some similarities were also found. In summary, these results suggest that T causes an extensive re-programing of liver transcriptome which is mainly an androgenic effect.

## Discussion

Preclinical and clinical studies suggest that hypothyroidism might cause endocrine and metabolic disturbances in the liver with features that mimic T and/or GH deficiencies (3). T plays relevant physiological effects on somatotropic-liver axis and liver composition and this is a primary organ for interactions between T and GH (13, 15). However, the complex effects of T, and its cooperation with GH, on gene expression profiling and lipid composition in hypothyroid rat liver are still misunderstood. In this study, we show that T replacement in hypothyroid male rats, and its interaction with pulsed GH, causes a significant reprogramming of transcriptome and lipid composition in the liver, and exerts a close interplay with somatotropic-liver axis, gender dimorphism and proteins involved in steroid and FA metabolism.

The positive cooperation between T and GH, that enhances their physiological effects on protein synthesis, IGF-I expression or fat oxidation, have been previously reported in hypopituitary men (13, 15); this cooperation mainly affects the liver rather than peripheral tissues (13, 15). In contrast, the lack of physiological cooperation between T and GH has been related to several liver-associated diseases (e.g., NAFLD) (24). This is in part because somatotropic-liver axis (e.g., GH and IGF-I) is a main regulator of intrahepatic lipid content (24) and the indirect effects of T in liver may result from interaction with the somatotropic-liver axis (13–15) and its influence to support a male-differentiated metabolism (5).

T and GH act as anabolic hormones and the positive cooperation between them on somatotropic-liver axis is clinically relevant (13–15). This was evident in previous studies in which boys with constitutional delay of body growth and development who exhibited lower serum levels of GH, IGF-I, and sex steroids compared to age-matched controls; this abnormal phenotype was reverted after T administration. Furthermore, adolescent boys with hypopituitarism required full replacement doses of both T and GH to normalize circulating IGF-I levels and reach maximal body growth (45–47). The negative effects of hypogonadism on somatotropic-liver axis were also evident in the present work performed in male hypothyroid rats, where the orchiectomy reduced body weight growth, circulating IGF-I or hepatic Igf-1 mRNA levels to greater extent than hypothyroidism without castration. These changes were partially restored upon T or pulsed GH replacement in TXOX rats, and they reached the levels of the age-matched euthyroid control group when these hormones were combined. This functional positive cooperation between T and GH on somatotropic-liver axis described in this study contrasts with the effects of E2 replacement in TXOX rats reported in a previous work from our lab, where the GH-induced increase in circulating IGF-I and IGFBP-3, lipid oxidation, and protein synthesis were inhibited by the estrogen (33). These effects are also contrary to those caused by E2 on somatotropic-liver axis in humans (48, 49). In the present work, the functional cooperation between T and GH was also observed on several negative regulators of GHR-STAT5b signaling pathway (50). Thus, decreased levels of circulating T and thyroid hormones in TXOX rats caused 80% reduction of Socs2 mRNA levels. This effect was not restored after T replacement whereas pulsed GH administration to TXOX rats upregulated Socs2 (and Cis) expression more than in the age-matched euthyroid INTACTSO control group. Despite GH-induced Cis mRNA remained unaffected, and Socs5 gene expression was induced by the combinatory treatment, T replacement in TXOX rats partially prevented the stimulative effects of GH on Socs2 mRNA expression. These findings suggest that T and GH could be cooperating through the prevention of Socs2 expression, the main negative regulator of longitudinal body growth (51). However, Socs3 gene expression was significantly upregulated by hypothyroidism itself and hypothyroidism plus castration in male rats (data not shown). Therefore, an increased expression of Socs3 (52, 53), a negative regulator of GHR-STAT5b signaling pathway in the liver, may also contribute to the inhibitory effect of hypothyroidism on somatotropic-liver axis described in the present work. Unlike T3, we observed that neither T nor GH were able to restore Socs3 mRNA expression in hypothyroid rat liver, thus supporting the hypothesis that thyroid hormones are key regulators of Socs3 gene expression. Our results also showed that T and GH further cooperated to modulate the transcription of male-differentiated genes in TXOX rat liver. Cyp2c11 gene expression is positively regulated by the male pattern of pituitary GH secretion through the GHR-STAT5b signaling pathway (54, 55), but it is not affected by T in the absence of pituitary hormones (54–56). In this study, hypothyroidism in male caused a 50% reduction of hepatic Cyp2c11 mRNA and its expression was abolished when castration was added to hypothyroidism. Pulsed GH replacement to TXOX rats induced Cyp2c11 expression but not Cyp2c12, a female-specific gene (5). Our microarray analysis also showed that the male-differentiated genes (5), α-2u-globulin and major urinary proteins were induced after T, pulsed GH administration, or combinatory treatment in TXOX rats. These findings support the efficacy of our hormonal replacements to maintain male-differentiated functions in TXOX rat liver. Paradoxically, we showed that T also increased the expression of female-specific Cyp2c12 gene to higher levels than in age-matched INTACTSO and TXOX-vehicle treated rats, and prevented the stimulative effects of pulsed GH on Cyp2c11 gene expression. This finding might be explained by an efficient conversion of T to E2 (4, 57). Similarly, T3 also induced Cyp2c12 in TXOX rat liver (data not shown), which supports the hypothesis that thyroid hormones could contribute to feminize the male rat liver. Regardless the molecular mechanisms, these findings point to a physiologic cooperation between T and GH to exert their full body growth-promoting effects and gender dimorphism in hepatic metabolism.

It has been reported that the negative effects of hypothyroidism on body growth development and lipid composition are associated, at least in part, with increased hepatic amino acid catabolism and ureagenesis (33, 58, 59). In contrast, the oral or parental administration of T can induce whole-body anabolic effects in human liver, by reducing the rate of protein oxidation through inhibition of nitrogen loss via the hepatic urea cycle (1, 2). Furthermore, T can functionally cooperate with GH to enhance its physiological effects on protein and energy metabolism, an effect that is developed mainly in the liver rather than in peripheral tissues (13, 15). Accordingly, the biological network analysis performed in our work showed that parenteral administration of T to TXOX rats induced a whole-body anabolic effect by reducing the rate of protein oxidation, and several genes involved in the metabolism of amino acids and urea were significantly downregulated (e.g., Otc, Asl, Gpt2). Similarly, GH downregulated genes involved in the catabolism of amino acids and ureagenesis, a positive effect on nitrogen balance previously described in other studies for hypophysectomized (60–62) and TXOX rats (33). Paradoxically, we observed that in the presence of T, GH administration to TXOX rats downregulated ureagenesis genes whereas several ones involved in amino acid biosynthesis were still upregulated. This apparent paradox is in agreement with previous findings (13, 15), where T was shown to promote protein anabolism by inducing amino acid biosynthesis rather than by inhibiting protein catabolism. Despite this, it should be pointed that a functional cooperation between T and GH must be developed in order to enhance the physiological effects on protein anabolism.

Energy demand can also be modulated by the regulation of lipogenesis that is often increased in situations of reduced energy expenditure, such as hypothyroidism and deficiency of GH-, E2- or T (2–4, 10). Clinical and animal studies have shown that T controls the expression of key regulatory proteins involved in glycolysis, glycogen synthesis and lipid metabolism that, in turn, regulate liver composition (2, 6, 7, 13). However, the molecular mechanisms by which T modulates these metabolic pathways have not been fully understood. Several effects of T on liver, particularly on glucose metabolism (e.g., increased insulin secretion and sensitivity, reduced hepatic glucose output), have been described to be direct and take place before any changes in body composition occur. However, indirect effects of T are more complex mainly due to its crosstalk with ERα- and GHR-mediated signaling pathways. The relevance of T aromatization to maintain glucose tolerance and insulin sensitivity via E2-ERα signaling, has been previously described in both male and female ArKO mice where the estrogen biosynthetic enzyme aromatase is inactive (33). In the present study, T replacement in TXOX rats increased the mRNA expression of several genes involved in glycolysis (e.g., Gapdh, Ldha, Pklr) and gluconeogenesis (e.g., Akr1a1) whereas phosphoenolpyruvate carboxykinase (Pck1) gene, a critical gene in liver gluconeogenesis (63), was downregulated. The ER gene expression in female rat liver is under multihormonal regulation by glucocorticoids, T3, GH, and T. However, GH plays a central role in this process where other hormones that influence the serum level of GH indirectly affect the expression of the ER gene (11, 12). However, our data demonstrated that neither T nor GH, nor their combinatory treatment increased the levels of ERα mRNA in TXOX rats at the end-time point. Despite this finding, we cannot rule out a transient regulation of ERα gene expression in TXOX rat liver and that the aromatization might be contributing to the effects of T in hepatic glucose metabolism. Contrary to this hypothesis, the impact of T replacement on lipid composition and lipid-related genes significantly differed from those previously described for E2. First, T, opposite to E2 (33), dramatically increased the levels of FFAs. Second, incorporation of pulsed GH completely abolished the effect of T on FFAs, but failed to cause any appreciable change on the effects of E2 (33). In the present study, we observed that the lipolytic effects of T in TXOX rat liver were turned into a lipogenic effect under pulsed pattern of GH administration. In contrast, a previous work from our group reported that the effect of E2 remained unaffected by GH, which seems to be always lipogenic with increased levels of TG (33). Third, compared to TXOX group, hepatic levels of 16:0, 18:0 and SFA in total lipids were reduced in the TPGH-treated group in the present work but this effect was not observed in E2GH-treated TXOX rats (33). When compared with INTACTSO or TXOXTP group, our results showed that GH treatment provoked a significant increase in arachidonic acid, but this effect was not observed in E2-treated counterparts (33). Fourth, in this study, T replacement in TXOX rats was not able to increase the transcription of hepatic genes linked to hepatic FA uptake, transport, activation or CHO removal nor PPARα target genes which, however, were positively regulated by E2 (33, 64). These findings indicate an absence of positive crosstalk between T and β/ω-oxidation of FA. Paradoxically, previous studies have reported that parenterally administered estrogens in women have either no effect or only very limited beneficial actions on lipid metabolism, whereas when they were orally administered, caused GH-IGF-1 signaling pathway resistance and increased circulating TGs levels (65). This apparent paradox could be explained by a direct effect of estrogen in the liver where it induces Socs2 expression which, in turn, inactivates GHR-STAT5-IGF-1 signaling. This mechanism increases TG uptake and storage in the liver (66–69). In contrast, our study showed that T replacement in TXOX rats enhanced the effects of T on GH-regulated somatotropic-liver axis, male dimorphism and genes involved in steroid and FA metabolism. Overall, these findings suggest that, instead of a consequence of T conversion to E2, the major effects of parental administration of T in liver are lipogenic and androgenic in nature.

Hypothyroidism in non-castrated male rats highly increased the hepatic level of total SFAs compared to euthyroid INTACTSO rats (33). This was a result of the enhanced levels of stearic acid (18:0) and reduced of MUFA 18:1n-7, thus indicating a reduction of stearoyl-CoA desaturase-1 (Scd1) activity. The accumulation of total SFAs was maintained upon castration but this effect was only apparent (i.e., when non-castrated hypothyroid rats were compared with the castrated hypothyroid group). Nevertheless, the apparent effect of castration was significantly revealed from our data when T replacement was combined with pulsed GH administration in TXOX rats. Interestingly, in this study we observed that GH alone restored total SFAs in TXOX rats to the same levels of INTACTSO animals, a situation where SFAs levels dropped and MUFA levels increased. The hepatic MUFA/SFA ratio was highest when T and GH were combined, which indicated that pulsed GH exerted a permissive action on the effects of T on MUFA synthesis. An increased MUFA/SFA ratio was linked to a significant reduction of stearic acid (18:0) and an elevation of MUFAs, particularly oleic acid (18:1n-9), which is the opposite to the effect provoked by hypothyroidism in male rats. Accordingly, T replacement in TXOX rats upregulated Serbp1c and Scd1, two key lipogenic genes (70), whose functional activation is evident by increased synthesis of MUFAs from SFAs, mainly oleate (18:1n9) and palmitoleate (16:1n7) from stearate (18:0) and palmitate (16:0), respectively (71, 72). In addition, our results showed that T induced the expression of other lipogenic genes, such as insulin induced gene-1 (Insig1) or FA desaturase 1 (delta-5 desaturase) (Fads1). Whereas Srebp1c is a main positive regulator of lipogenesis, Insig1 could inhibit biosynthesis of cholesterol by blocking the cleavage of Srebps and inducing 3-hidroxi-3-metil-glutaril-CoA reductase (HMGCR) protein degradation (73). The Srebp1-Sdc1-MUFAs pathway may be regulated by the lipid-activated transcription factors PPARβ/δ and liver X receptor α (LXRα) (74). Accordingly, other studies have reported that PPARβ/δ mediates Scd1 transcription and further increases endogenous MUFAs synthesis, thus resulting in a positive loop of regulation that protects liver from accumulation of toxic lipids by converting SFAs into MUFAs (74, 75). In contrast, MUFAs might negatively regulate Scd1 gene expression by blocking SREBP1c cleavage (76). Additionally, Scd1 gene could be indirectly regulated by LXRα-SREBP1c signaling pathway, and directly regulated by LXRα (77, 78) since a functional LXR response element has been identified in the mouse Scd1 promoter, and Scd1 gene remained induced upon LXR activation even in the absence of Srebp-1c (77, 78). If any of this FA-dependent activation of the mentioned pathways might contribute to increase MUFA/SFA ratio in T-treated TXOX rat liver, still deserves further analysis.

Most of the biochemical and physiological actions of PUFAs depend on the conversion of 18C dietary sources to 20C or 22C LCPUFA and subsequent metabolism to lipid mediators, and they could bind directly, or through their derivatives (i.e., eicosanoids), to lipid sensor receptors and transcription factors (e.g., PPARs, LXRα, Srebp1c) (79, 80). In our study, T administration to TXOX rats increased Fads1 transcription, a gene with a pivotal role in the availability of LCPUFA with relevant biological activities such as arachidonic acid (20:4n-6) and EPA (20:5n-3), and DHA (22:6n-3) (81). The levels of arachidonic acid and DHA in INTACT and TXOX rats were three-to-four times higher in total lipids than in neutral lipids (data not shown), indicating their preferential location within membrane phospholipids. Noteworthy, in the present work we demonstrated that T abolished the positive effects of GH on the biosynthesis of essential LCPUFA (i.e., T alone or combined with GH failed to modify EPA and DHA contents). In this case, the crosstalk between T and GH was evident because DHA levels were higher when GH was administered alone. Despite an apparent role of T, the functional role of gonadal hormones on DHA and arachidonic acid levels in hypothyroid rat liver was relevant because they were higher and lower in non-castrated hypothyroid rats than in castrated-hypothyroid rats, respectively. T replacement in TXOX rats downregulated Fatty Acid Binding Protein 7 (Fabp7) and Elongation of very long chain fatty acids protein 7 (Elovl7), which could reduce the availability of FAs (e.g., linoleic acid for oxidation) or the elongation of LCPUFA, respectively. Remarkably, Fabp7 might govern the transcriptional activity of PPAR by targeting the LCPUFA to nucleus to regulate PPAR-dependent transcription (82). Whether this mechanism contributes to regulate the effects of T- or GH-regulated LCPUFA synthesis and PPAR-dependent transcription still deserves extensive research. PPARα is a pivotal transcriptional regulator of genes involved in FA β/ω-oxidation (83). However, our results showed that T replacement in TXOX rats, unlike E2, for which has been shown a marked positive crosstalk with PPARα pathway (33), did not increase the transcription of PPARα gene itself or many PPARα target genes involved in the β/ω-oxidation of FAs (e.g., Cte-1, Cpt-2, Fasd6, Fasd2, Ech1, Fgf21, Cyp4A1, Cyp4A3) nor hepatic genes linked to hepatic FA uptake, transport, activation or cholesterol removal (e.g., Cd36, Ldlr, Acsl4, Fatp5, ApoC2, Mttp, Cyp7a1). These findings indicated the absence of marked positive crosstalk between the effects of T on hepatic FA composition and PPARα.

Overall, our findings demonstrate the complex interactions between T and GH to achieve hepatic FA composition. Despite GH and T act as anabolic hormones, they modulate lipogenesis in tissue-dependent manner. GH promotes lipolysis, and prevents lipogenesis in adipose tissue, to increase the availability of circulating FA for energy expenditure (84). However, DHT could increase visceral fat mass associated with TG accumulation in mice a treatment that increased expression of Srebp2 and Fas, and decreased the inactivating phosphorylation of acetyl-CoA Carboxylase (Acc) (85). In our study, T replacement in TXOX rats increased FFA levels while reducing TG, and modified the ratio SFA/MUFA, affecting the unsaturation index of hepatocytes. The reduction in hepatocyte TG was paralleled by their increase in circulating levels. These findings agree with the observation that T increased Scd-1 gene expression, an enzyme that catalyzes the synthesis of MUFAs, mainly oleate and palmitoleate, two major components of membrane phospholipids, TG, cholesteryl esters and wax esters (86). Moreover, T upregulated lipogenic genes (i.e., Srebp1, Fads1, Faah), while decreased Fabp7 and FA receptor (Far4) expression, supported the lipogenic effect of T in liver. In addition to a direct AR-dependent effect in liver, the effects of T on lipid metabolism might result from its interaction with GH (13–15). Our study shows that the mechanism through which combination of T and pulsed GH administration induces a lipogenic phenotype in TXOX rat liver is complex but mobilization of SFAs from FFA to TG might be involved. In contrast to T, GH increased hepatic TG, but decreased FFA whereas the levels of DG were notably increased by GH and/or TP treatment. Pulsed GH administration to TXOX induced several genes linked to unsaturated FAs biosynthesis (e.g., Fasd1, Elvol5, Fabp5), which might also contribute to lipid composition in GH- or TPGH-treated TXOX rat. Alternatively, given their lipolytic actions (87), GH might increase flux of FFAs to the liver and contribute to lipid content in TXOX liver. Accordingly, co-administration of T and GH increased both hepatic and circulating TG levels to a greater extent than either hormone alone. The increase in hepatic TG by combination of T and GH also suggests a synergistic lipogenic effect which was supported by the increased expression of Scd1 and lipogenic genes (e.g., Me, malate dehydrogenase) (88). Furthermore, TPGH treatment enhanced the expression levels of hepatic genes linked to FA metabolism and resulted in increased MUFA levels (mainly 18:1n9) from total lipids, compared to TP and GH treatments, individually. TPGH also induced lipogenic genes (i.e., Srebp1c, Me, G6pdh, Pgdh, Hmgcr, Fas, Acc) and Acacα, a gene that encodes the enzyme that catalyzes the rate-limiting step in LCFA synthesis, Elovl5 and Scd1, which contributes to the synthesis of DHA and MUFA (88). Moreover, T-mediated induction of Insig1, a gene that prevents Srebp activation, was inhibited by GH and this might contribute to lipogenic effect of combination of T and GH (i.e., TPGH). The combination of T and GH also upregulated the oxysterol binding protein (Osbp) gene in TXOX liver which may limit uptake and synthesis of CHO through transcriptional inhibition of Ldlr, Hmg synthetase, and Hmgcr (89, 90). In accordance, TP plus GH treatment reduced hepatic CHO, with concomitant increase of cholesteryl esters, compared to GH monotherapy, indicating an antagonistic hormonal interaction. Since only intrahepatic cholesteryl esters but not CHO levels increased after this combinatory treatment, cholesteryl esters synthesis might happen with a simultaneous CHO biosynthesis. Although we did not detect changes in the expression level of the Hmgcr or Acat genes in TPGH-treated rats, these findings did not discard posttranslational modifications of enzymes in the cholesteryl esters biosynthesis cycle. Alternatively, because T replacement increased the formation of phosphatidylcholine in TXOX animals, but reduced when it was combined with GH, it can be argued that free lysophosphatidylcholine (LPC) resulting from LCAT-mediated PC hydrolysis might be rapidly re-esterified to phosphatidylcholine, giving rise to increased contents of both cholesteryl esters without a reduced phosphatidylcholine. These two hypotheses are not necessarily exclusive but can occur synergistically to increase cholesteryl esters. Interestingly, T replacement modified the hepatic contents of anionic phospholipids. T increased levels of PS and reduced phosphatidylinositol and sphingomyelin whereas these effects were reversed by pulsed GH administration. This effect was not observed in a previous study form our lab carried out in E2-treated TXOX rats (33). These results suggest an influence on phospholipid biosynthesis from phosphatidic acid through modification of enzyme activity/expression in the CDP-activated 1,2-diacylglycerol (CDP-DG) and Kennedy pathways (91). Accordingly, our differential gene expression analysis showed that GH increased the mRNA level of choline kinase-α (Ckα), the first enzyme in the Kennedy pathway and responsible for *de novo* synthesis of phosphatidylcholine (91), an effect which was abolished in the presence of T. However, phosphocholine cytidylyltransferase (CTP) gene that catalyzes a rate-limiting and regulated step in the CDP-choline pathway for the synthesis of phosphatidylcholine and phosphatidylcholine-derived lipids, and choline dehydrogenase were upregulated by GH despite the presence of T. These observations are in consonance with the fact that hepatocyte phosphatidylcholine levels (and secondarily phosphatidylethanolamine and total polar lipids) were substantially reduced in rats receiving the combined treatment with T and GH. Therefore, our results showed that T replacement in TXOX rats prevented the positive effects of GH on Ckα gene expression, which might result in the CDP-choline cycle inhibition and in elevated diacylglycerol levels in TPGH rat liver compared to INTACTSO, TXOX, TXOXTP, or TXOXGH groups. Finally, these data support the hypothesis that the modulation of the CDP-choline cycle by T and GH might affect the dynamics of membrane remodeling and the distribution of lipid-related metabolites in cellular pathways, and may contribute to the combined effects of the mentioned hormones on liver transcriptome.

In summary, this study highlights the influence of T on lipid composition and transcriptome and its impact on GH-regulated endocrine and metabolic functions in hypothyroid liver and reveals key clinical implications of the interplay between T and GH. These findings support the hypothesis that liver-targeted T therapy may open up a new approach to achieve whole-body anabolism both alone and in combination with pulsed GH administration. Furthermore, T may be superior to nonaromatizable androgens in inducing a complex spectrum of direct estrogen-mediated and GH-mediated effects on somatotropic-liver axis as well as in liver metabolism and composition.

## Data availability statement

The datasets presented in this study can be found in online repositories. The names of the repository/repositories and accession number(s) can be found in the article/**Supplementary Material.**

## Ethics statement

All animal experiments were conducted in accordance with Spanish and European Union (EU) animal care guidelines and with approved protocol by the Animal Care and Use Committee of the University of Las Palmas de Gran Canaria (OEBA-ULPGC 2006/07824).

## Author contributions

LF-P: Conceptualization, Formal analysis, Funding acquisition, Investigation, Supervision, Writing – original draft, Writing – review & editing. BG: Conceptualization, Formal analysis, Funding acquisition, Investigation, Supervision, Writing – original draft, Writing – review & editing. CR: Investigation, Supervision, Writing – review & editing. JC-G: Funding acquisition, Investigation, Supervision, Writing – review & editing. IG: Formal analysis, Investigation, Methodology, Writing – review & editing. JD: Data curation, Formal analysis, Investigation, Methodology, Writing – review & editing. AC: Investigation, Supervision, Writing – review & editing. DI-G: Conceptualization, Formal analysis, Funding acquisition, Investigation, Methodology, Supervision, Writing – original draft, Writing – review & editing. MD: Conceptualization, Data curation, Formal analysis, Funding acquisition, Investigation, Supervision, Writing – original draft, Writing – review & editing.

## Supplementary material

Supplementary Figure S1 Procedures and treatments performed to obtain the different experimental rat models. The euthyroid testis-intact (INTACT) and hypothyroid (TX) rat models were generated as described in Material and Methods. INTACT rats were sham-operated (SO) to obtain euthyroid testis-intact controls (INTACTSO). TX rats were orchiectomized or SO to obtain hypothyroid-orchiectomized (TXOX) or hypothyroid testis-intact (TXSO) models, respectively **(A)**. TXOX rats were treated with vehicle (TXOX group) or testosterone propionate (TP; TXOXTP group), plus growth hormone (GH; TXOXGH and TXOXTPGH groups), triiodothyronine (T3; TXOXT3 and TXOXTPT3 groups) or T3 plus GH (TXOXT3GH group) as described in Material and Methods **(B)**.

Supplementary Table 1 Gene abbreviations: 3-hydroxyisobutyrate dehydrogenase (Hibadh); ATP citrate lyase (Acly); ATP synthase subunit b, mitochondrial-like (ATP5F1); NADH:ubiquinone oxidoreductase subunit B3 (Ndufb3); NADH:ubiquinone oxidoreductase subunit B8 (Ndufb8); ST6 beta-galactoside alpha-2,6-sialyltransferase 1 (St6gal1); UDP glucuronosyltransferase 2 family, polypeptide B35 (Ugt2b35); UDP-glucose 6-dehydrogenase (Ugdh); aldehyde dehydrogenase 1 family, member A1 (Aldh1a1); aldehyde oxidase 1 (Aox1); aldo-keto reductase family 1 member A1 (Akr1a1); alpha-methylacyl-CoA racemase (Amacr); argininosuccinate lyase (Asl); bile acid CoA:amino acid N-acyltransferase (Baat); choline dehydrogenase (Chdh); cysteine dioxygenase type 1 (Cdo1); cytochrome c oxidase subunit 8A (Cox8a); diacylglycerol O-acyltransferase 2 (Dgat2); dihydropyrimidinase (Dpys); glyceraldehyde-3-phosphate dehydrogenase (Gapdh); hexose-6-phosphate dehydrogenase (H6pd); histidine ammonia lyase (Hal); hydroxysteroid (17-beta) dehydrogenase 2 (Hsd17b2); isocitrate dehydrogenase 3 (NAD+) beta (Idh3B); ketohexokinase (Khk); lactate dehydrogenase A (Ldha); malate dehydrogenase 1 (Mdh1); ornithine carbamoyltransferase (Otc); paraoxonase 3 (Pon3); phosphoenolpyruvate carboxykinase 1 (Pck1); platelet-activating factor acetylhydrolase 1b, regulatory subunit 1 (Pafah1b1); proline dehydrogenase 1 (Prodh1); pyruvate kinase, liver and RBC (Pklr); secretory blood group 1 (Sec1); squalene epoxidase (Sqle); tryptophan 2,3-dioxygenase (Tdo2); epoxide hydrolase 1 (Ephx1); glutathione S-transferase pi 1 (Gstp1); SEC22 homolog B, vesicle trafficking protein (Sec22b); eukaryotic translation elongation factor 1 alpha 1 (Eef1a1); eukaryotic translation elongation factor 1 gamma (Eef1g); secretion associated, Ras related GTPase 1B (Sar1b); ribosomal protein L17 (Rpl17); ribosomal protein L27a (Rpl27a); ribosomal protein L28 (Rpl28); ribosomal protein L4 (Rpl4); ribosomal protein S12 (Rps12); ribosomal protein S15 (Rps15); ribosomal protein S15a (Rps15a); fatty acid desaturase 1 (Fads1); stearoyl-CoA desaturase (Scd); ribosomal protein L27a (Rpl27a); ribosomal protein L28 (Rpl28); ribosomal protein L4 (Rpl4); ribosomal protein S12 (Rps12); ribosomal protein S15 (Rps15); ribosomal protein L17 (Rpl17); ribosomal protein S15a (Rps15a).

Supplementary Table 2 Gene abbreviations: ATP citrate lyase (Acly); malate dehydrogenase 1 (Mdh1); malate dehydrogenase 2 (Mdh2); phosphoenolpyruvate carboxykinase 1 (Pck1);pyruvate carboxylase (Pc); pyruvate dehydrogenase (lipoamide) beta (Pdhb); succinate dehydrogenase complex subunit C (Sdhc); succinate-CoA ligase ADP-forming beta subunit (Sucla2); aconitase 1 (Aco1); fumarate hydratase (Fh); succinate-CoA ligase, GDP-forming, beta subunit (Suclg2); acyl-CoA oxidase 3, pristanoyl (Acox3); acyl-CoA synthetase long-chain family member 4 (Acsl4); carnitine palmitoyltransferase 2 (Cpt2); fatty acid binding protein 1 (Fabp1); fatty acid desaturase 2 (Fads2); peroxisome proliferator-activated receptor delta (Ppard); phosphoenolpyruvate carboxykinase 1 (Pck1); retinoid X receptor alpha (Rxra); stearoyl-coenzyme A desaturase 3 (LOC681458); solute carrier family 27 member 5 (Slc27a5); stearoyl-CoA desaturase (Scd); sterol carrier protein 2 (Scp2); acyl-CoA dehydrogenase, C-4 to C-12 straight chain (Acadm); acyl-CoA oxidase 1 (Acox1); Cyp8b1; enoyl-CoA hydratase and 3-hydroxyacyl CoA dehydrogenase (Ehhadh); Cyp2b2; Cyp2c22; Cyp2c24; Cyp2c6v1; Cyp2c7; glutathione S-transferase alpha 5 (Gsta5); glutathione S-transferase omega 1 (Gsto1); kynurenine aminotransferase 1 (Kyat1); microsomal glutathione S-transferase 1 (Mgst1); UDP glucuronosyltransferase 1 family, polypeptide A6 (Ugt1a6); Cyp2c23; Cyp2e1; Cyp3a18; glutathione S-transferase mu 2 (Gstm2); glutathione S-transferase pi 1 (Gstp1); hydroxysteroid 11-beta dehydrogenase 1 (Hsd11b1); UDP glucuronosyltransferase 2 family, polypeptide B1 (Ugt2b1); catechol-O-methyltransferase (Comt); Cyp2b2; Cyp2d2; steroid 5 alpha-reductase 1 (Srd5a1); UDP glucuronosyltransferase 1 family, polypeptide A6 (Ugt1a6); Cyp11a1; hydroxysteroid (17-beta) dehydrogenase 2 (Hsd17b2); mitochondrial ribosomal protein L34 (Mrpl34); 40S ribosomal protein S6-like (LOC100911372); mitochondrial ribosomal protein L32 (Mrpl32); ribosomal protein L13 (Rpl13); ribosomal protein L14 (Rpl14); ribosomal protein L17 (Rpl17); ribosomal protein L19 (Rpl19); ribosomal protein L22 (Rpl22); ribosomal protein L23 (Rpl23); ribosomal protein L32 (Rpl32); ribosomal protein L34 (Rpl34); ribosomal protein L37a (Rpl37a); ribosomal protein L7 (Rpl7); ribosomal protein S14 (Rps14); ribosomal protein S15a (Rps15a); ribosomal protein S16 (Rps16); ribosomal protein S17 (Rps17); ribosomal protein S20 (Rps20); ribosomal protein S24 (Rps24); ribosomal protein s25 (Rps25); ribosomal protein S27 (Rps27); ELOVL fatty acid elongase 5 (Elovl5); fatty acid desaturase 1 (Fads1); acyl-CoA dehydrogenase, C-4 to C-12 straight chain (Acadm); acyl-CoA oxidase 1 (Acox1); enoyl-CoA hydratase and 3-hydroxyacyl CoA dehydrogenase (Ehhadh); aldolase, fructose-bisphosphate B (Aldob); argininosuccinate synthase 1 (Ass1); enolase 1 (Eno1); glutamic-oxaloacetic transaminase 2 (Got2); glyceraldehyde-3-phosphate dehydrogenase (Gapdh); pyruvate carboxylase (Pc); pyruvate kinase, liver and RBC (Pklr); pyruvate kinase, muscle (Pkm); serine hydroxymethyltransferase 2 (Shmt2); triosephosphate isomerase 1 (Tpi1); aconitase 1 (Aco1); cystathionine gamma-lyase (Cth); ornithine carbamoyltransferase (Otc); alanine-glyoxylate aminotransferase (Agxt); betaine-homocysteine S-methyltransferase(Bhmt); choline dehydrogenase (Chdh); glyoxylate and hydroxypyruvate reductase (Grhpr); alanine-glyoxylate aminotransferase 2 (Agxt2); dimethylglycine dehydrogenase (Dmgdh); pipecolic acid and sarcosine oxidase (Pipox); ATP synthase, H+ transporting, mitochondrial F1 complex, delta subunit (Atp5d); ATP synthase, H+ transporting, mitochondrial F1 complex, alpha subunit 1, cardiac muscle (Atp5a1); ATP synthase, H+ transporting, mitochondrial Fo complex, subunit C3 (subunit 9) (Atp5g3); cytochrome c-1 (Cyc1); NADH:ubiquinone oxidoreductase core subunit V1 (Ndufv1); ATP synthase, H+ transporting, mitochondrial F1 complex, beta polypeptide (Atp5b); cytochrome c oxidase subunit 6C (Cox6c); NADH:ubiquinone oxidoreductase subunit A4 (Ndufa4); ATP synthase, H+ transporting, mitochondrial Fo complex, subunit C1 (subunit 9) (Atp5g1); NADH dehydrogenase (ubiquinone) 1 alpha subcomplex 10 (Ndufa10); cytochrome c oxidase subunit 8A (Cox8a); cytochrome c oxidase subunit 4i1 (Cox4i1); succinate dehydrogenase complex subunit C (Sdhc); cytochrome c, somatic (Cycs); G protein subunit alpha i3 (Gnai3); NADH:ubiquinone oxidoreductase subunit B10 (Ndufb10); PTEN induced putative kinase 1 (Pink1); voltage-dependent anion channel 2 (Vdac2); aldehyde dehydrogenase 9 family, member A1 (Aldh9a1); aldo-keto reductase family 1 member A1 (Akr1a1); lactate dehydrogenase A (Ldha); retinol saturase (Retsat); RPE65, retinoid isomerohydrolase (Rpe65); aldehyde dehydrogenase 1 family, member A1 (Aldh1a1); coagulation factor XII (F12); complement C8 alpha chain (C8a); complement C8 beta chain (C8b); fibrinogen alpha chain (Fga); fibrinogen gamma chain (Fgg); coagulation factor XI (F11); coagulation factor XIII B chain (F13b); complement C1q B chain (C1qb); complement C1s (C1s); complement component 4 binding protein, beta (C4bpb); kallikrein B1 (Klkb1); apolipoprotein E (Apoe); LDL receptor related protein 1 (Lrp1); mitogen activated protein kinase 1 (Mapk1); acyl-CoA dehydrogenase, very long chain (Acadvl); adiponectin receptor 2 (Adipor2); CCAAT/enhancer binding protein alpha (Cebpa); activating transcription factor 4(Atf4); cell division cycle 42 (Cdc42); phospholipase A2, group XVI (Pla2g16); aldo-keto reductase family 7 member A3 (Akr7a3); glutathione S-transferase alpha 5 (Gsta5); glutathione S-transferase omega 1 (Gsto1); microsomal glutathione S-transferase 1 (Mgst1); UDP glucuronosyltransferase 1 family, polypeptide A6 (Ugt1a6); flavin containing monooxygenase 9 (Fmo9); flavin containing monooxygenase 5 (Fmo5); pyrophosphatase (inorganic) 2 (Ppa2); REST corepressor 1 (Rcor1); Sec61 translocon alpha 1 subunit (Sec61a1); Sec61 translocon alpha 2 subunit (Sec61a2); signal recognition particle 19 (Srp19); SRP receptor alpha subunit (Srpra); SEC62 homolog, preprotein translocation factor (Sec62).

Supplementary Table 3 Abbreviations for lipid classes: CE = cholesterol esters; CHO = cholesterol; DG = diacylglycerols; FFA = free (unesterified) fatty acids; LPC = lysophosphatidylcholine; PC = phosphatidylcholine; PE = phosphatidylethanolamine; PG = phosphatidylglycerol; PLE = PE-plasmalogens; PI = phosphatidylinositol; PS = phosphatidylserine; SM = sphingomyelin; TG = triacylglycerols; TNL= total neutral lipids; TPL= total polar lipids.

Supplementary Table 4 Abbreviations for total lipids: SFA = saturated fatty acids; MUFA = monounsaturated fatty acids; PUFA = polyunsaturated fatty acid; UI = unsaturation index; PI = peroxidation index.
