# Supplementary figures and images for: Transcriptomic and lipid profiling analysis reveals a functional interplay between testosterone and growth hormone in hypothyroid liver
Source: Front Endocrinol (Lausanne). 2023 Dec 8;14:1266150. doi: 10.3389/fendo.2023.1266150 (PMC10748415; doi:10.3389/fendo.2023.1266150)

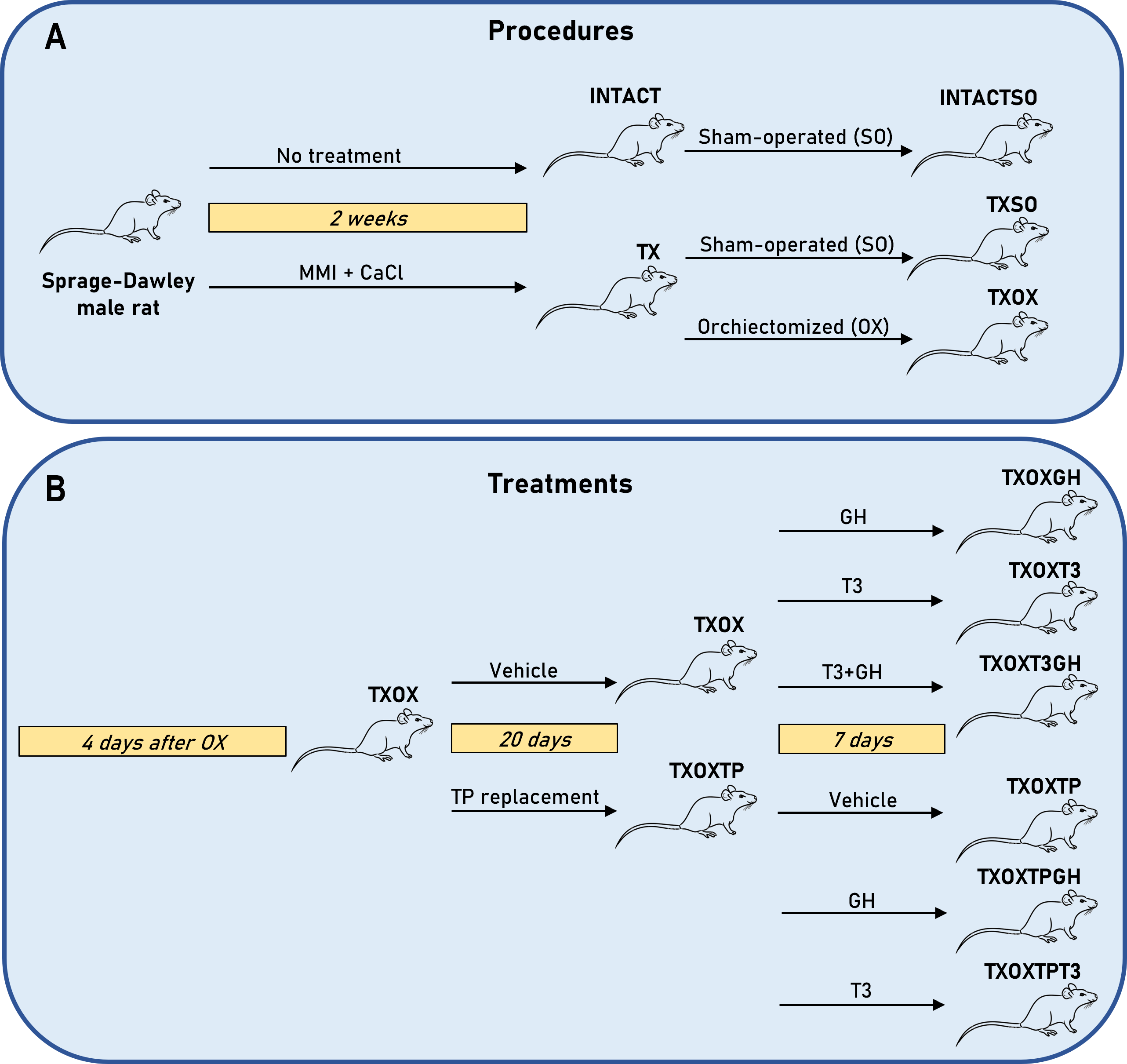

Supplement: Supplementary Figure S1 — Procedures and treatments performed to obtain the different experimental rat models. The euthyroid testis-intact (INTACT) and hypothyroid (TX) rat models were generated as described in Material and Methods. INTACT rats were sham-operated (SO) to obtain euthyroid testis-intact controls (INTACTSO). TX rats were orchiectomized or SO to obtain hypothyroid-orchiectomized (TXOX) or hypothyroid testis-intact (TXSO) models, respectively (A). TXOX rats were treated with vehicle (TXOX group) or testosterone propionate (TP; TXOXTP group), plus growth hormone (GH; TXOXGH and TXOXTPGH groups), triiodothyronine (T3; TXOXT3 and TXOXTPT3 groups) or T3 plus GH (TXOXT3GH group) as described in Material and Methods (B). [file Image_1.tif]
